# Upregulation of the Coagulation Factor VII Gene during Glucose Deprivation Is Mediated by Activating Transcription Factor 4

**DOI:** 10.1371/journal.pone.0040994

**Published:** 2012-07-27

**Authors:** Katherine R. Cronin, Thomas P. Mangan, Josephine A. Carew

**Affiliations:** 1 Department of Research, VA Boston Healthcare System, West Roxbury, Massachusetts, United States of America; 2 Department of Medicine, Harvard Medical School, Boston, Massachusetts, United States of America; University of Hong Kong, Hong Kong

## Abstract

**Background:**

Constitutive production of blood coagulation proteins by hepatocytes is necessary for hemostasis. Stressful conditions trigger adaptive cellular responses and delay processing of most proteins, potentially affecting plasma levels of proteins secreted exclusively by hepatocytes. We examined the effect of glucose deprivation on expression of coagulation proteins by the human hepatoma cell line, HepG2.

**Methodology/Principal Findings:**

Expression of coagulation factor VII, which is required for initiation of blood coagulation, was elevated by glucose deprivation, while expression of other coagulation proteins decreased. Realtime PCR and ELISA demonstrated that the relative percentage expression +/− SD of steady-state *F7* mRNA and secreted factor VII antigen were significantly increased (from 100+/−15% to 188+/−27% and 100+/−8.8% to 176.3+/−17.3% respectively, p<0.001) at 24 hr of treatment. The integrated stress response was induced, as indicated by upregulation of transcription factor *ATF4* and of additional stress-responsive genes. Small interfering RNAs directed against ATF4 potently reduced basal *F7* expression, and prevented *F7* upregulation by glucose deprivation. The response of the endogenous *F7* gene was replicated in reporter gene assays, which further indicated that ATF4 effects were mediated via interaction with an amino acid response element in the *F7* promoter.

**Conclusions/Significance:**

Our data indicated that glucose deprivation enhanced *F7* expression in a mechanism reliant on prior ATF4 upregulation primarily due to increased transcription from the *ATF4* gene. Of five coagulation protein genes examined, only *F7* was upregulated, suggesting that its functions may be important in a systemic response to glucose deprivation stress.

## Introduction

Hepatocytes produce most of the coagulation proteins found in plasma, and for many coagulation proteins are the only cell type known to do so. Though their average concentrations and half-lives differ, all coagulation proteins are present in trace amounts in plasma and have comparatively short half-lives. Since sufficient concentrations of all the coagulation proteins are necessary for hemostasis, constitutive expression of the genes encoding them is required to maintain adequate plasma concentrations. Hepatocytes must frequently alter their patterns of metabolic gene expression in response to oscillations in nutrient availability and the consequent hormonal shifts engendered by normal behaviors such as feeding or fasting, and by changing levels of activity. In addition, hepatocytes are responsible for the secretion many specialized plasma proteins, such as serum albumin and apolipoproteins, as well as coagulation proteins. However, the question of whether the rates of coagulation protein transcription and biosynthesis are consistent, or whether they fluctuate in response to environmental conditions, has largely been unexplored.

Coagulation FVII (FVII) plays a pivotal role in blood coagulation, yet has the lowest average plasma level among the vitamin K-dependent coagulation proteins (500 ng/ml or ∼10 nM) as well as the shortest half-life (3–6 hr). Because of these properties, variations in the extent of hepatic FVII expression could be particularly important with regard to hemostasis. FVII is the zymogen of a glycoprotease essential for the initial reaction of blood coagulation, and is indispensable for both hemostasis and thrombosis. [1], [2]To initiate blood coagulation, FVII must interact with its transmembrane receptor, tissue factor (TF), be converted to its catalytically active form, FVIIa, and then must proteolytically activate its physiological substrates, the zymogens of coagulation factors X (FX) and IX (FIX). [1] These activated coagulation proteases, in turn, participate in a complex series of reactions with additional coagulation proteins such as factors VIII and V (FVIII and FV) on the membrane surfaces of activated platelets or other cells, resulting in the proteolyic activation of prothrombin to thrombin. The coagulation pathway culminates with the thrombin-mediated conversion of soluble fibrinogen to insoluble fibrin to form the structure of the blood clot. (reviewed in [3], [4]) In contrast, additional coagulation proteins, such as Protein C and Protein S, are important in counter-reactions that limit the overall extent of blood coagulation. [5], [6].

The interaction between FVII/FVIIa and TF is tightly regulated by the relative amounts and disparate locations of each component, as well as by their reciprocal requirements for functional activation. The plasma concentration of FVII is low, with 1% of the total amount estimated to be the activated form (reviewed in [7]). However, FVIIa has negligible catalytic activity against FIX and FX when in solution; only when bound to TF in an appropriate cell membrane context does FVIIa attain its full catalytic ability. Zymogen FVII as well as FVIIa can interact with TF. After FVII binds TF, it is rapidly converted to FVIIa either autocatalytically, by the FVII activating protease (FSAP) or by trace amounts of other activated coagulation proteins. [2], [8] Although TF is more abundant than FVII/FVIIa, being expressed on the membranes of many cell types throughout the body, it is found almost exclusively outside the vasculature (reviewed in [9]). Furthermore, it too exists in an inactive (or “encrypted”) form. [10] Encrypted TF has the ability to bind, but not to support the catalytic activity of, FVII/FVIIa. [11] The activation (“decryption”) of TF has been associated with influx of calcium ions into TF-expressing cells [12], the local loss of membrane lipid asymmetry [13], the dissociation of TF dimers [10] and disulfide exchange at the Cys186–Cys209 bond in the external domain of TF. [14], [15] These physical and biochemical barriers limit the majority of productive TF–FVII/FVIIa interactions to the time and place of a vascular injury.

The importance of having sufficient amounts of FVII and TF for hemostasis has been demonstrated through characterization of patients with FVII deficiency (TF deficiency has never been documented in humans [16]), as well as through study of knockout mice in which one or the other gene has been completely or partially silenced. Patients homozygous for deleterious promoter mutations reducing transcription of the FVII gene (F7) [17], [18], [19], [20], [21], although rare, have been diagnosed following severe bleeding episodes that usually first occur during infancy. The phenotype of the F7 knockout mouse is reminiscent of that of patients with severe FVII deficiency. If gestation takes place in a dam expressing murine FVII from at least one normal allele, the FVII −/− pups develop properly but experience fatal internal hemorrhages shortly after birth. [22], [23] Ablation of the TF gene is an embryonic lethal defect [24], [25], but normal development takes place in mice expressing very low TF levels, ∼1% of the typical level. Hemostasis is apparently normal in this “low-TF” mouse, except in situations where extensive bleeding must be controlled. [24].

The endoplasmic reticulum (ER) is an important organelle within all eukaryotic cells, and its proper functioning is especially significant for differentiated cell types such as hepatocytes that must meet continual heavy metabolic and biosynthetic demands. In hepatocytes, the roles of the ER include the maintenance of intracellular calcium levels, the production of membrane lipids and cholesterol, the metabolism of glycogen, the detoxification of both xenobiotics and the byproducts of normal metabolism such as lactate and ammonia, as well as the synthesis, initial folding and post-translational modification of an array of specialized transmembrane and secreted proteins. As secreted proteins, FVII and other coagulation factor proteins must traverse the hepatocyte ER during biosynthesis. Many circumstances, both physiologic and pharmacologic, have been shown to compromise the ability of the ER to optimally handle protein synthesis, and as a cell type with high protein secretory activity, hepatocytes may be particularly sensitive to conditions that trigger cellular stress responses. [26] A common repercussion of such insults is a sharp reduction in global protein translation, which lessens the cell’s energetic burden at a time when resources are limited. Translational inhibition comes about through the reversible phosphorylation of the eukaryotic translation initiation factor 2 alpha (eIF2α) at a single residue, impairing its ability to interact with GTP and blocking binding of the initiator methionyl-transfer RNA to ribosomes, thus inhibiting new protein translation. [27], [28], [29], [30], [31].

At least four kinases are known to be capable of phosphorylating eIF2α, thereby repressing global protein synthesis in response to different types of stress: these are HR1 (EIF2AK1), PKR (EIF2AK2), PERK (EIF2AK3), and GCN2 (EIF2AK4). HR1 is known to be activated by oxidative stress, PKR by viral infection, PERK by damage to the ER or by accumulation of misfolded proteins within it, and GCN2 by amino acid deprivation or UV irradiation [29], [31], [32], [33], [34] In addition to the immediate reduction in global protein synthesis, gene expression patterns are altered in stressed cells to bring about a longer-term response to the stressor. Paradoxically, phosphorylated eIF2α also enhances the selection of the appropriate open reading frame in the messenger RNA encoding the transcription factor ATF4 (activating transcription factor 4) and so preferentially increases ATF4 translation at a time when overall protein translation is inhibited. The resultant increase in ATF4 functionality is succeeded by the upregulated expression of numerous ATF4 target genes, many but not all of which also encode transcription factors. [35], [36], [37], [38], [39], [40], [41], [42], [43], [44], [45], [46] Collectively, this eIF2α-mediated mechanism has been referred to as the “integrated stress response” [47].

Conditions that interfere directly with ER functioning, causing unfolded nascent proteins to accumulate within it, activate additional stress response pathways. Three ER transmembrane proteins–an eIF2α kinase mentioned above (PERK, or protein kinase RNA-dependent-like endoplasmic reticulum kinase), ATF6 (activating transcription factor 6) and IRE1α (inositol requiring enzyme 1 alpha)–mediate the three branches of the ER stress response (reviewed in [48]). In homeostasis, the ER luminal domains of each of these proteins is thought to be bound by GRP-78 (glucose related protein 78, also known as BiP) an abundant ER chaperone protein, and this interaction maintains them in their inactive states. When improperly folded proteins accumulate within the ER, there is an increased need for GRP-78 to act as a chaperone and so it dissociates from PERK, ATF6 and IRE1α. For each of these “sensor proteins”, its dissociation from GRP-78 is the prerequisite for activation. [49] PERK is activated by dimerization and autophosphorylation [50] early in the stress response. Inactive ATF6 is translocated from the ER to the Golgi apparatus following its dissociation from GRP-78, where it is cleaved by SP1 and SP2 (the resident site-1 and site-2 proteases) to release the active ATF6α transcription factor. [51] After translocation to the nucleus, ATF6α, in conjunction with the transcription factor NF-Y, upregulates expression of target genes that include the chaperone proteins *GRP-78*, *GRP-94* and *PDI* (protein disulfide isomerase), as well as of transcription factor *XPB1* (X-box binding protein 1). Although production of ATF6α is fairly rapid, the subsequent upregulation of its target genes requires some time to become apparent. Finally, when IRE1α is released from GRP-78, it dimerizes and is autophosphorylated, activating an internal endoribonuclease. [50] Two functions for the IRE1α ribonuclease have been reported: the degradation of mRNAs encoding secreted or membrane proteins, which further reduces the protein synthetic burden of the ER, [52] and atypical splicing of XPB1 mRNA. Translation of unspliced XBP1 mRNA produces a DNA binding protein without a transactivation domain. When the IRE1α ribonuclease removes a 26-base pair intron from XPB1 mRNA, the reading frame is altered to encode a fully functional transcription factor. [53], [54], [55] Many target genes of XPB1 overlap with those of ATF6α, while others promote the degradation of misfolded proteins. This third branch of the ER stress response is the last to be fully implemented, since a significant level of XBP1 mRNA is not attained prior to upregulation of the XBP1 gene by ATF6α. [54] The co-ordinated activation of these pathways thus limits global protein synthesis by blocking the translation of most proteins, producing additional protein chaperones to assist with the folding of immature proteins within the ER lumen, and enhancing the degradation of mRNAs and severely misfolded proteins. Although higher-level activation of stress response pathways, especially if sustained over time, will lead to apoptosis, these mechanisms taken together are well suited to resolve short-term or lower-intensity stresses that cause unanticipated difficulties with protein synthesis. [56].

Investigations of cellular stress responses have largely been directed towards detailed understanding the intracellular pathways involved, and have seldom explored the effect of stresses upon the expression of cell-type specific secreted proteins. The expected slowdown in global protein processing characteristic of stress responses, together with possible reductions in mRNA and nascent protein stability, suggest that production of all secretory proteins would be curtailed. In this study, we have examined whether stress triggered by glucose deprivation influenced expression of five coagulation protein genes–those encoding the vitamin K-dependent FVII, FX, prothrombin and protein S, as well as FVIII, in the human hepatoma cell line, HepG2.

To our knowledge, no previous investigation has analyzed the extent to which nutritional stress impinges on constitutive expression of blood coagulation proteins. Our results indicated that 24 hr glucose deprivation activated multiple stress response pathways in HepG2 cells, and was associated with reduced mRNA levels transcribed from the *F10*, *F2* (prothrombin), *PROS1* (protein S) and *F8* genes. Conversely, however, we found that the expression of the *F7* gene was enhanced at both the mRNA and the secreted protein levels by glucose deprivation. Focusing on *F7* transcriptional upregulation, we further demonstrate that it occurs in an ATF4-dependent manner, mediated by increased transcription of the *ATF4* gene and direct interaction of ATF4 protein with a composite binding element within the proximal promoter of the *F7* gene.

## Results

### Glucose Deprivation Differentially Affects Expression of Coagulation Protein Genes

**Figure 1:**
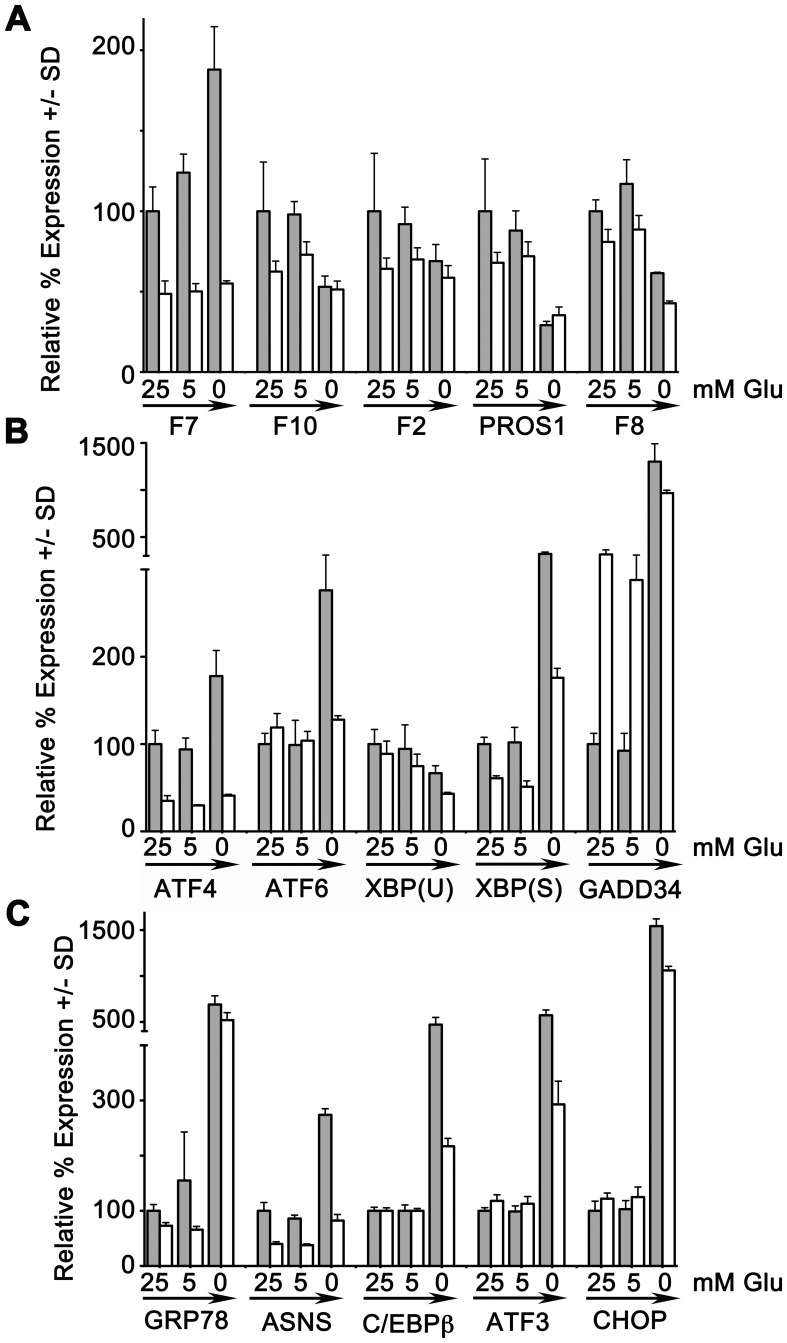
Glucose deprivation and coagulation factor mRNA expression. Replicate cultures of HepG2 cells were transfected with negative control siRNA (grey bars) or ATF4 siRNA #1 (white bars), then cultured in media with 25 mM, 5 mM, or 0 mM glucose for 24 hr. RNA was analyzed by qRT-PCR for expression of amplicons as shown. For each amplicon, average expression at 25 mM glucose with the negative control siRNA was considered 100% and other values are shown as % expression +/− SD relative to that. N = 3 per group. Panel **A**, coagulation factor genes; panels **B** and **C**, stress responsive genes. At 0 mM glucose, expression was significantly increased for *F7, ATF4, ATF6, XBP1(S), GADD34, GRP-78, ASNS, C/EBPβ, ATF3* and *CHOP* (all p<0.001). Please note difference in scale between panel A and panels B and C. ATF4 siRNA blocked all increases (all p<0.005) except for *GRP-78*, *GADD34 and CHOP.* Expression of *FX, F2 and PROS1* were significantly decreased at 0 mM glucose (all p<0.001) and were not significantly affected by ATF4 siRNA.

To examine the ability of glucose deprivation to influence the expression of coagulation factor genes, HepG2 cells were incubated in standard culture media supplemented with 1% fetal bovine serum and glucose at high, standard, or no-glucose conditions (25 mM, 5 mM and 0 mM glucose, respectively). It was anticipated that glucose deprivation would activate the integrated stress response and thus increase the amount of functional ATF4 transcription factor present in the cells. Therefore, this initial experiment was performed upon cells that had been transfected either with a negative control siRNA or with a validated siRNA directed against ATF4. After 24 hr of treatment, total RNA was isolated, converted to cDNA, and tested by quantitative reverse-transcriptase realtime PCR as shown in Figure 1, to determine the relative levels of mRNA encoding the genes of interest: several coagulation factor genes, the ATF4 gene and a number of its target genes, and additional known stress-inducible genes. A complete list of the genes examined, their genebank accession numbers, and the Taq-Man gene expression assay numbers used, are given in Table S1.

With regard to expression of the coagulation factor genes (Figure 1A), the data demonstrate that in cells transfected with the negative control siRNA, the *F10*, *F2* (prothrombin), *PROS1* and *F8* genes were each significantly downregulated at the mRNA level by glucose deprivation. In contrast, the expression of *F7* steady-state mRNA was significantly upregulated by glucose deprivation, from 100+/−15% to 188+/−27%. Glucose deprivation for 24 hr also increased expression of the *ATF4* gene at the mRNA level (Figure 1B). The steady-state *ATF4* mRNA was elevated nearly two-fold (from 100+/−15.7% to 178+/−29.3%). These data suggested that *ATF4* transcription was elevated, while the increased expression of several recognized, stress-inducible target genes of ATF4 (*ASNS*, *C/EBPβ*, *ATF3*) (Figure 1C) implied that ATF4 protein levels and transcriptional capacity had also been elevated during glucose deprivation.

Increased mRNA levels were also observed for two “growth arrest and DNA damage-inducible” or GADD genes: *CHOP* (*GADD153*), an inhibitory member of the C/EBPβ transcription factor family, and *GADD34,* an accessory protein for the serine/threonine-protein phosphatase PP1 which is needed to reverse the stress-induced repression of protein synthesis mediated by phosphorylated eIF2α. [57] In addition, increased mRNAs encoding the transcription factors *ATF6* and spliced *XBP1(S)* despite a decrease in total *XPB1(U)* mRNA, and of the ER chaperone protein *GRP-78*, were also observed. (Figure 1B, 1C) These data indicated that all ER stress response pathways, not only the integrated stress response, had been triggered in the cells by glucose deprivation.

The inclusion of ATF4 siRNA reduced the mRNA levels of some genes in this panel, indicating the ones whose basal and/or induced expression relied upon functional ATF4. Glucose deprivation in the presence of ATF4 siRNA blocked basal ATF4 mRNA expression as well as *ATF4* upregulation during glucose deprivation, and also prevented the upregulation of *C/EBPβ*, *ATF3*, and *ASNS*. In contrast, ATF4 siRNA had only minimal influence on the induction of *GADD34*, *GRP-78* and *CHOP*. Interestingly, ATF4 siRNA blocked *ATF6* induction, and partially prevented the increased splicing of *XBP1(S)* during glucose deprivation as well. Crosstalk has been previously reported between the integrated stress response and these other branches of the ER stress response. [58], [59].

ATF4 siRNA did not significantly affect the basal expression, or prevent the downregulation during glucose deprivation, of the coagulation protein genes *F10*, *F2*, *PROS1* and *F8*. Of the coagulation factor genes tested, only *F7* responded to ATF4 siRNA, and its responses were strong and were observed at all three glucose concentrations. The *F7* steady-state mRNA levels were halved by inclusion of ATF4 siRNA in comparison to the negative control siRNA, at both 25 mM and 5 mM glucose. Most notably, inclusion of ATF4 siRNA completely eliminated the increase in *F7* mRNA anticipated at 0 mM glucose. The response of *F7* to the ATF4 siRNA resembled more closely that of *ASNS* and of the *ATF4* gene itself, than it did even of two other known ATF4 target genes, *ATF3* and *C/EBPβ*. Taken together, the data of Figure 1 implied a previously unrecognized role for ATF4 in endogenous expression of *F7*, and suggested as well that *F7* is a transcriptional target of *ATF4* during the integrated stress response. Further experiments were then conducted to examine these topics in more detail.

### Thapsigargin and Glucose Deprivation Trigger Similar Stress Responses, but have Distinct Effects on *F7* Expression

**Figure 2:**
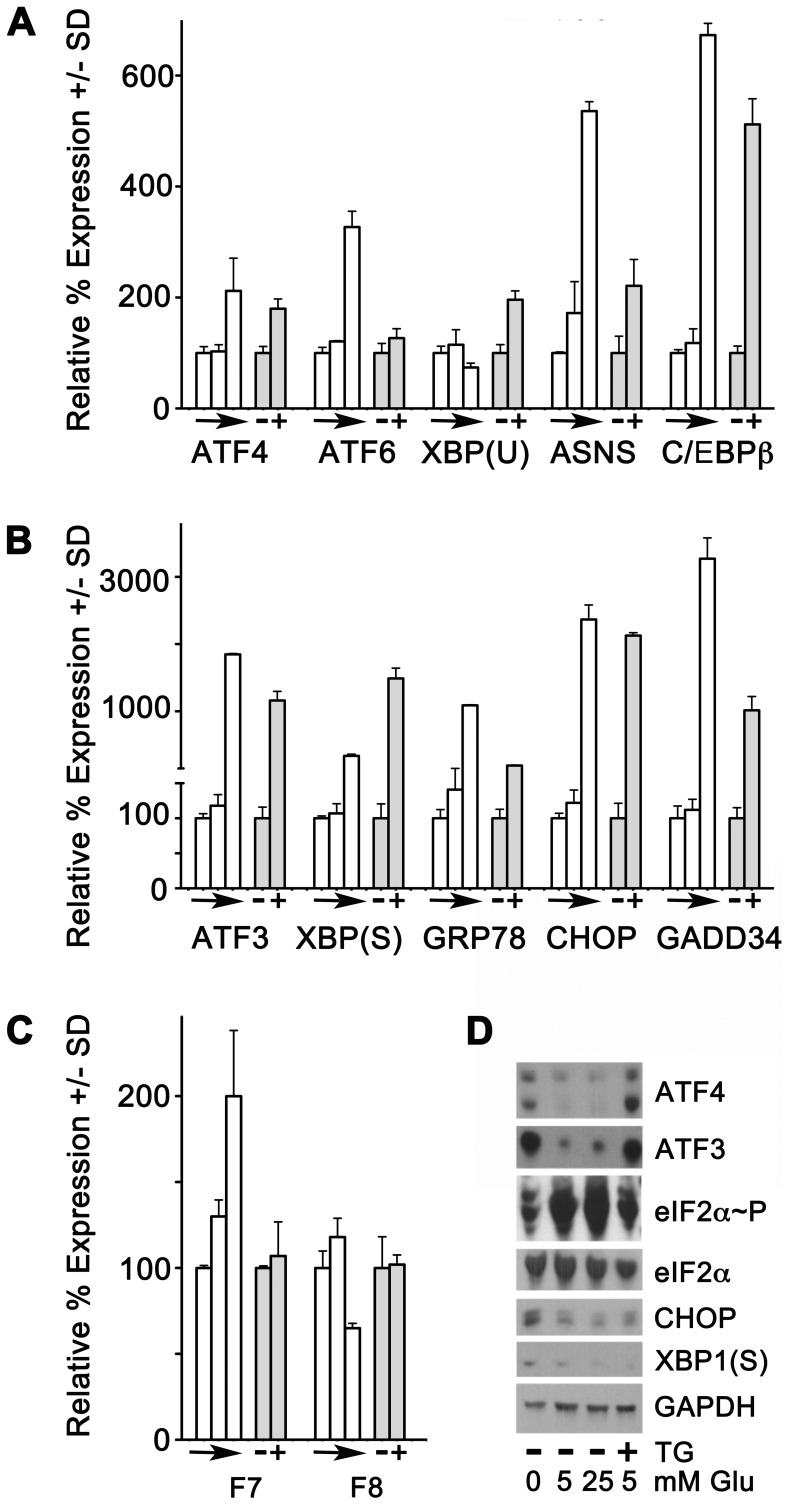
Thapsigargin versus glucose deprivation and F7 mRNA expression. (**A-C**) HepG2 cells were treated in parallel for 24 hr in medium with 25 mM, 5 mM or 0 mM glucose (white bars, with the direction of arrows indicating decreasing concentration) or for 6 hr at 5 mM glucose without (-) or with (+) 500 nM TG (grey bars), and qRT-PCR was performed as described. Expression at 25 mM glucose and without thapsigargin were considered 100% expression for each amplicon; other groups were expressed as a percentage +/− SD relative to this. N = 3/group. Glucose deprivation significantly increased expression of *ATF4, ATF6, ASNS, C/EBPβ, XBP1(S), GRP78, CHOP, GADD34,* and *F7* (all p<0.001). TG did likewise (all p<0.001), except for *ATF6* and *F7* amplicons. *F8* was downregulated by glucose deprivation but unaffected by TG. (D) Western blot of whole-cell extracts of cells treated with glucose and/or TG as shown, above the lanes, for detection of the proteins shown at the right.

Thapsigargin (TG), an irreversible inhibitor of the ER-membrane calcium ATPase, interferes with normal ER functioning and provokes strong and rapid activation of all branches of the ER stress response. [45], [49], [51], [60] A TG concentration of 500 nM and a time of 6 hr were chosen for the comparison to 24 hr glucose deprivation for HepG2 cells, since in each instance *ATF4* expression was roughly doubled. In Figure 2, the ability of TG to affect gene expression was compared to that of glucose deprivation. Panels A and B provide the results of qRT-PCR performed in parallel for a number of stress-inducible genes. As in glucose deprivation (Figure 1, and confirmed here) *ATF4* and its target genes *ATF3*, *C/EBPβ*, and *ASNS*, as well as *GADD34 GRP-78*, *XBP1(U)* and *XBP1(S)* were all significantly upregulated by TG. *ATF3, ASNS*, *GADD34* and *GRP-78* were increased to a greater extent by glucose deprivation than by TG; *CHOP* and *C/EBPβ* were increased to approximately the same extent; and *XBP1(U)* and *XBP1(S)* were increased to a greater extent by TG than by glucose deprivation. Only glucose deprivation increased expression of *ATF6.* Finally, as shown in panel C, it was striking that TG had no effect at all on expression of the two coagulation factor genes tested: *F7* was not upregulated, nor was *F8* downregulated, as they were by glucose deprivation.

In Figure 2 panel D, protein expression for a subset of these stress-responsive genes (ATF4, ATF3, CHOP, and XBP1(S)) were investigated, along with pan- and phospho-eIF2α. Both glucose deprivation and TG treatment increased the detectable amounts of ATF4 and ATF3, while CHOP and XBP1(S) were increased by glucose deprivation at the protein level, corroborating their expression at the mRNA level. While the amounts of total eIF2α remained fairly constant, we noted that higher amounts of phospho-eIF2α were seen at 25 mM and 5 mM glucose than at 0 mM glucose and 500 nM TG. This result appeared contradictory, since the greatest detection of phospho-eIF2α might have been expected under the strongest stress conditions. However, we also noted that mRNA expression of *GADD34*, which assists PP1 to dephosphorylate eIF2α and thereby permits global protein translation to resume, was also much higher under glucose deprivation (3271+/−309%) or TG treatment (1015+/−205%) than at 25 mM or 5 mM of glucose (100+/−17.6% and 112+/−15.2%, respectively). Thus we hypothesized that eIF2α had been extensively phosphorylated (or was being repeatedly yet transiently phosphorylated) in glucose-deprived and TG-treated cells, but that its phosphorylation status was being reversed through induction of GADD34.

**Figure 3:**
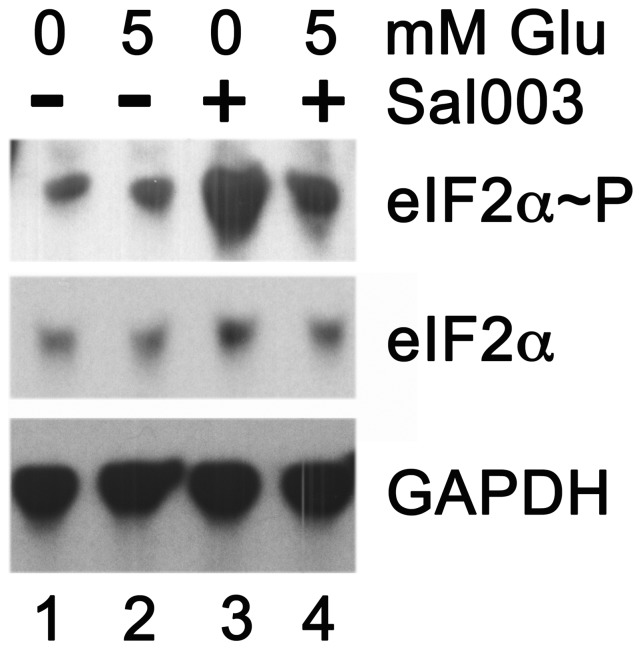
Phosphorylation of eIF2α during glucose deprivation. Western blots of extracts from HepG2 cells incubated for 6 hr in media having either 0 mM or 5 mM glucose, and without (-) or with (+) 10 µM Sal003 as shown above lanes. Antibodies against phospho-eIF2α (eIF2α∼P), total eIF2α and GAPDH as a loading control, were used as indicated to the right of each blot.

To test this hypothesis, we performed an additional experiment in which cells were cultured for 6 hr with 5 mM or 0 mM glucose, in the absence versus the presence of the specific PP1 inhibitor, Sal003. [61] Because the eIF2α dephosphorylation mechanism is inhibited, any phospho-eIF2α generated under glucose deprivation will accumulate when Sal003 is included. The Western blot comparing eIF2α and phospho-eIF2α under these conditions is shown in Figure 3. Approximately comparable amounts of phospho-eIF2α were detected in cells cultured with 5 mM or 0 mM glucose in the absence of Sal003 (compare lanes 1 and 2). At 5 mM glucose, the detectable phospho-eIF increased slightly when 10 µM Sal003 was present during the incubation period (compare lanes 2 and 4). However, the detectable phospho-eIF2α increased robustly in cells cultured with 0 mM glucose and 10 µM Sal003 (compare lanes 1 and 3). It is interesting to note that even under the standard culture conditions, eIF2α was phosphorylated to a minor degree, and hence was capable of enhancing the translation of ATF4 mRNA somewhat. These data indicate that phosphorylation of eIF2α happens to a greater extent during glucose deprivation, implying that enhanced transcription of the *ATF4* gene and preferential translation of ATF4 mRNA cooperate in the upregulation of *F7* and other ATF4 target genes.

### FVII is Secreted in an ATF4-dependent Manner during Glucose Deprivation

**Figure 4:**
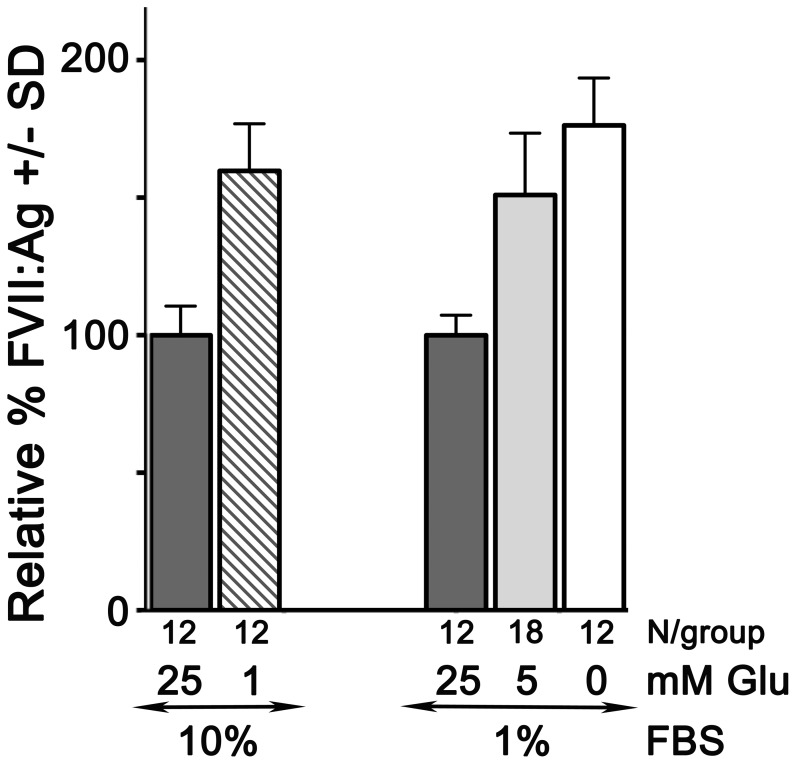
Glucose concentration affects secreted FVII antigen levels. HepG2 cells were cultured for 24 hr in media supplemented with 10% or 1% fetal bovine serum and either high (25 mM), standard (5 mM), or low/no (1 mM or 0 mM) glucose. The concentration of secreted FVII antigen, expressed as ng per ml, was determined by ELISA. For each experimental set, the average amount secreted by cells in 25 mM glucose was considered 100%, and amounts secreted at the lower glucose concentrations were expressed as percentages +/− SD relative to that. The number of replicates assayed at each condition is shown below the bars. Reducing the concentration of glucose significantly increased the amount of FVII secreted for each experimental set (p<0.001).

To determine the extent to which the elevation in *F7* mRNA in response to glucose deprivation was reflected in translation and secretion of FVII antigen, HepG2 cells were incubated in media with glucose levels ranging from 25 mM to 0 mM, and the amount of FVII secreted into the culture medium over a 24 hr period was measured by ELISA (Figure 4). Glucose at 25 mM, 5 mM, and 1 mM were employed to mimic the plasma glucose levels in unregulated diabetics, and in non-diabetics during fed and fasted states, respectively. [62] Though unlikely to occur in physiological contexts, the glucose deprivation condition (0 mM glucose), was included as an established method to strongly provoke cellular stress responses, [41], [42], [45], [46] the efficacy of which we had confirmed, above. Low glucose concentrations, either 1 mM or 0 mM, significantly increased the amount of FVII antigen detected in the culture medium. For cells in media containing 10% serum, reduction of glucose from 25 mM to 1 mM increased the amount of secreted FVII from 100+/−10.5% to 159.7+/−17.2% (p<0.001). For cells in media containing 1% serum, reduction from 25 mM to 0 mM glucose increased FVII antigen from 100+/−8.8% to 176.3+/−17.3% (p<0.001). In these experiments, any insulin present was contributed by the serum component of the media and was constant under a given condition. Since glucose limitation increased the secreted FVII regardless of the amount of serum present, this effect was independent of insulin or any other serum component. For convenience, subsequent experiments were performed in media containing 1% serum.

As a control to examine whether glucose deprivation treatments were survivable, we have measured both the cell number and the overall metabolic activity by a colorimetric MTS (3-(4,5-dimethylthiazol-2-yl)-5-(3-carboxymethoxyphenyl)-2-(4-sulfophenyl)-2H-tetrazolium) assay in cells cultured for 24 hr in media with 1% serum containing either 5 mM or 0 mM glucose. (Figure S1). While there was a notable reduction in metabolic activity, from 100+/−10% to 13+/−1.4%, associated with glucose deprivation, this was not accompanied by a reduction in cell number. Thus we concluded that the *F7*/FVII induction provoked by the glucose deprivation was likely to be part of an adaptive response to a severe but survivable stress condition.

### ATF4 Increases *F7* Expression by Direct and Indirect Mechanisms

As described above, an important early event in cellular stress responses is preferential translation of ATF4 mRNA at the correct ORF to produce the functional transcription factor. This may also be accompanied by elevated transcription of the ATF4 gene as noted by other investigators and as we observed for both glucose deprivation and TG (Figures 1 and 2) [35], [41], [45], [47], [56]. Whether ATF4 directly affected transcription of the F7 gene, either under basal conditions or during the stress response, was not previously known.

**Figure 5:**
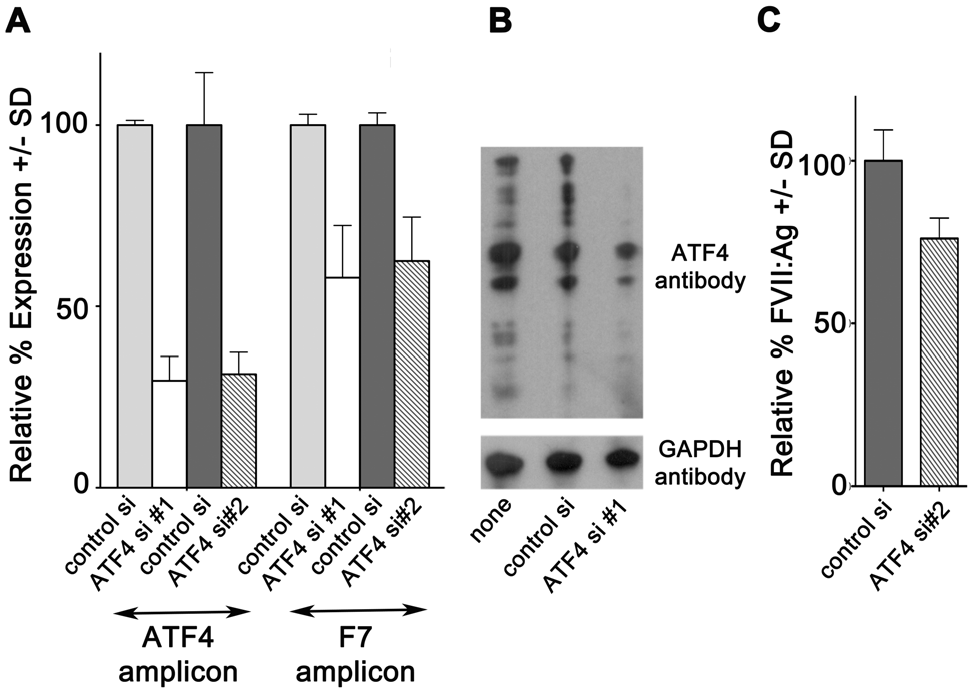
ATF4 siRNA blocks basal F7 expression. A. siRNA directed to either the 5' UTR (ATF4 si#1, white bars) or the 3' UTR (ATF4 si#2, striped bars) of the human *ATF4* gene were introduced into HepG2 cells cultured in media with 5 mM glucose. Parallel cultures were transfected with the same amount of negative control siRNA (control si, light and dark grey bars, respectively). Relative expression of *ATF4* and *F7* are graphed as a percentage +/− SD of the expression seen with negative control siRNA, which was considered 100%. For ATF4 si#1, N = 5 for ATF4 amplicon and N = 10 for F7 amplicon; for ATF4 si #2, N = 6 per group. All p<0.001 for both ATF4 siRNAs. **B.** Whole-cell extracts prepared from HepG2 cells transfected with negative control siRNA (control si), 5' UTR-ATF4 siRNA (ATF4 si#1) or without siRNA (none) and cultured 48 hr in media with 5 mM glucose. Replicate aliquots of extracts, 4 µg/lane, were separated on SDS-PAGE and Western-blotted in parallel with antibody to ATF4 (upper panel) or to GAPDH (lower panel) as loading control. **C.** Conditioned media collected from cells following transfection with negative control siRNA (dark grey bars) or ATF4 si#2 (striped bars) for 48 hr in media with 5 mM glucose, analyzed for FVII antigen by ELISA. N = 18/group, p<0.001.

The results of our initial experiment suggested that ATF4 influenced *F7* transcription under both basal circumstances and during glucose deprivation. To investigate this more closely, we first employed an RNA interference strategy to assess the degree to which ATF4 influences ambient *F7* expression in unstressed cells. As shown in Figure 5A, the endogenous ATF4 expressed by HepG2 cells under the standard culture condition of 5 mM glucose was independently knocked down by two small interfering RNAs (siRNAs), one directed to the 5' UTR and the other to the 3' UTR, of the *ATF4* gene.

The data of Figure 5A demonstrate that each ATF4 siRNA was effective in reducing the steady-state ATF4 mRNA to ∼30% of the level seen in cells transfected with the negative control siRNA. At the same time, each ATF4 siRNA decreased F7 steady-state mRNA to ∼60% of its level in cells transfected with the negative control siRNA. The western blot of Figure 5B for ATF4 protein expression and the ELISA of Figure 5C for FVII antigen secretion demonstrate that transfection with ATF4 siRNA caused a reduction in the ATF4 and F7 protein levels reflecting their respective mRNA reductions. The amount of detectable ATF4 protein, assessed by Western blotting of whole-cell extracts, declined dramatically post-introduction of ATF4 siRNA. Similarly, introduction of ATF4 siRNA reduced the cumulative amount of FVII secreted into conditioned media as assessed by ELISA, from 100+/−9.5% to 76.1+/−6.3%, p<0.001. These data clearly indicate that even under the standard growth conditions, unstressed HepG2 cells were expressing endogenous ATF4, and that the extent of ATF4 expression was positively correlated with that of F7.

**Figure 6:**
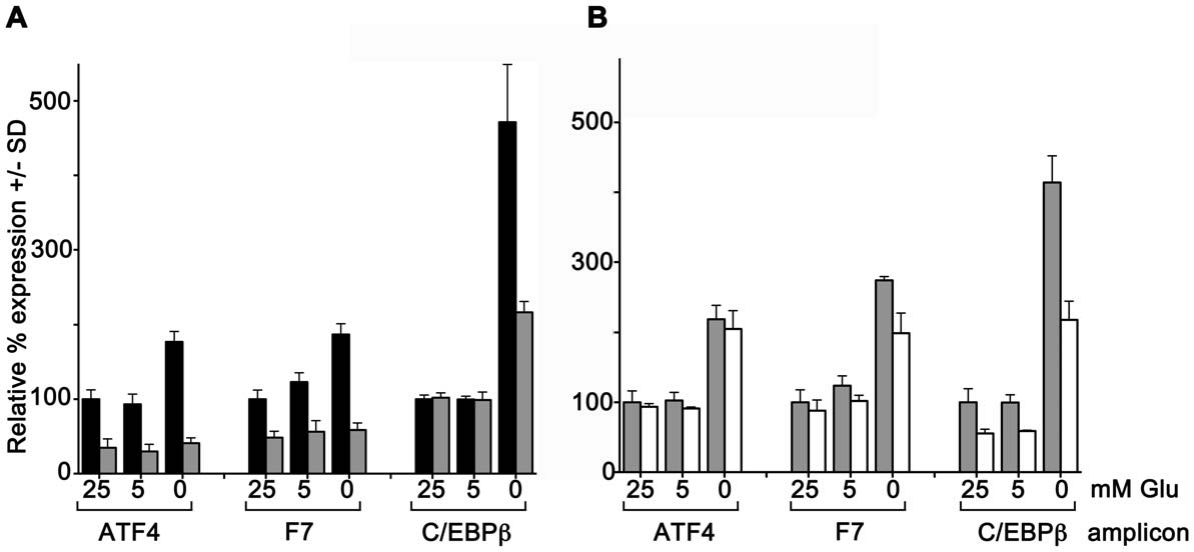
ATF4 siRNA and C/EBPβ siRNA differentially block F7 induction. A. Replicate cultures of HepG2 cells were transfected with ATF4 siRNA #2 (grey bars), or negative control siRNA (black bars), then cultured with 25 mM, 5 mM, or 0 mM glucose for 24 hr. qRT-PCR was done for expression of ATF4, F7, and C/EBPβ amplicons. For each amplicon, average expression +/− SD at 25 mM glucose with negative control siRNA was considered 100% and other values are shown relative to that. N = 3 per group, p<0.001 at all glucose concentrations for ATF4, F7 and ASNS amplicons, and for the C/EBPβ amplicon at 0 mM glucose. **B.** Experiment as in panel A, except that C/EBPβ siRNA (white bars) was used with negative control siRNA (grey bars). C/EBPβ siRNA had no effect on ATF4 amplicon expression at all glucose concentrations, but significantly blocked expression and induction of C/EBPβ at all glucose concentrations and induction of F7 at 0 mM glucose (p<0.001), N = 3 per group.

We have previously demonstrated that F7 expression is modulated in response to insulin, via a promoter element that interacts with transcriptionally activating and inhibiting isoforms of C/EBPβ. [63] Because C/EBPβ is known to be upregulated by ATF4 during nutritional stress [36], [42], and because the data of Figures 1 and 2 suggest that C/EBPβ expression is ATF4 dependent during glucose deprivation, our data could not differentiate whether the increase in transcription of the F7 gene was a direct effect of ATF4 upregulation, or an indirect effect mediated by ATF4-dependent upregulation of the C/EBPβ gene. To address whether or not fluctuating *ATF4* expression influenced *F7* directly or indirectly, we assessed the individual effects of C/EBPβ siRNA versus ATF4 siRNA on *F7* expression during glucose deprivation. (Figure 6) The data presented in both panels of Figure 6 again corroborated the upregulation of the *ATF4*, *F7*, and *C/EBPβ* genes by glucose deprivation in the presence of negative control siRNA. Reduction of glucose from 25 mM to 5 mM had negligible effects on expression of all three genes. In contrast, the further reduction from 5 mM to 0 mM glucose significantly increased their relative expression levels. For the *ATF4* and *F7* genes, steady-state mRNA levels were approximately doubled by glucose deprivation, while for *C/EBPβ* they were approximately quadrupled. Introduction of ATF4 siRNA prior to glucose deprivation (Figure 6A) once again completely prevented upregulation of both *ATF4* and *F7*, and significantly reduced but did not block upregulation of *C/EBPβ*. In contrast, while introduction of C/EBPβ siRNA prior to glucose deprivation did interfere with the extent of both *C/EBPβ* and *F7* upregulation, it did not prevent induction of *ATF4* by glucose deprivation (Figure 6B). Further, since ATF4 knockdown completely blocked *F7* induction by glucose deprivation, but *C/EBPβ* knockdown did not, we concluded that the response of the *F7* gene to glucose deprivation was taking place partially but not solely via an intermediate, C/EBPβ-dependent mechanism, and partially via a direct ATF4-dependent mechanism.

### Glucose Deprivation Drives F7 Reporter Gene Expression through the AARE Motif

Stress-induced increases in expression of many ATF4 target genes are known to be mediated by interactions of transcriptional regulatory proteins with an “amino acid response element”, or AARE. AAREs function as enhancers and have been identified within 5′ and 3′ regulatory regions, as well as within introns, of several stress-inducible genes. [36], [37], [38], [39], [40], [41], [42], [43], [44], [45], [46] AAREs have also been shown to interact with transcription factors of the basic leucine-zipper (b-zip) superfamily, of which both the CCAAT enhancer binding proteins (C/EBPs) and the activating transcription factors (ATFs) and are members. [44], [64], [65] Our previous work had shown that the *F7* promoter includes an AARE between positions −8 and +1 of the F7 promoter (where +1 represents the first base of the initiator methionine codon of the FVII structural gene), and that C/EBPβ affects *F7* expression via this element. [63]Thus we hypothesized that the glucose-deprivation dependent *F7* upregulation might be mediated, at least in part, via the AARE.

**Figure 7:**
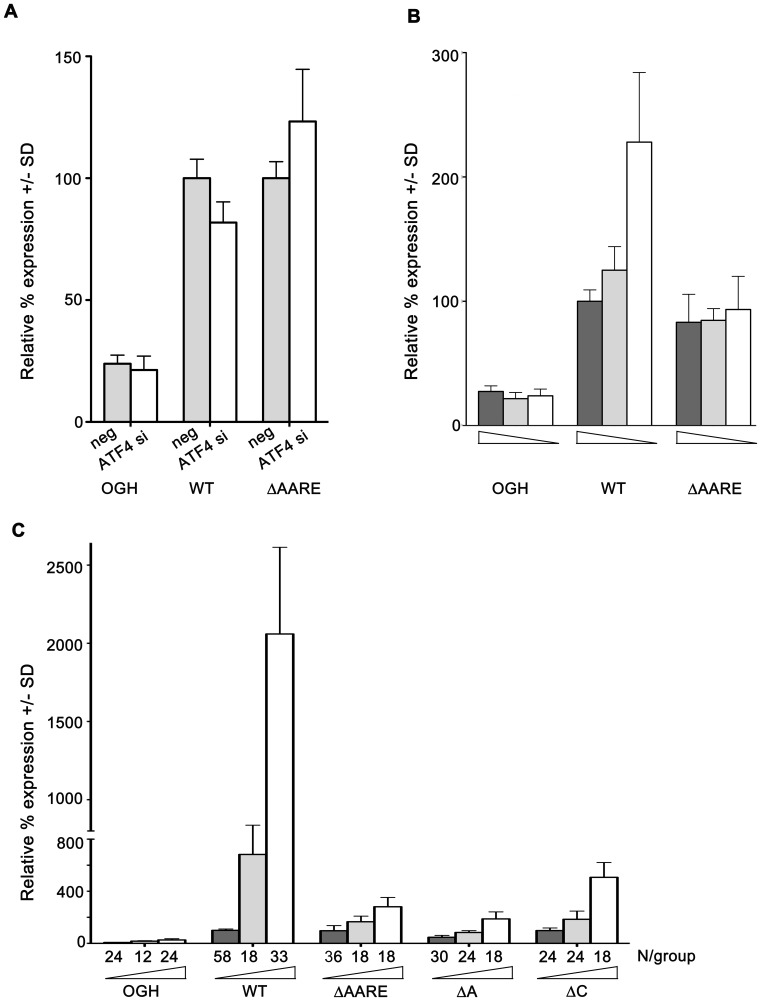
AARE mediates F7 glucose deprivation response. A. HepG2 cells were transfected with promoterless or reporter vectors OGH (N = 6/group), AARE-WT or ΔAARE (N = 12/group), along with either negative control siRNA or ATF4 si#1, then cultured at 5 mM glucose for the final 24 hr before harvest. Reporter expression is shown relative to that of AARE-WT vector in the presence of control siRNA, which was considered 100%. Effect of ATF4 siRNA on expression from AARE-WT vector only, p<0.001. **B.** HepG2 cells were transfected with reporter vectors and incubated in medium containing 25 mM glucose (dark grey bars), 5 mM glucose (light grey bars) or 0 mM glucose (white bars), with diminishing glucose concentration depicted by wedges. Glucose deprivation increased expression from WT reporter (N = 12; p<0.001), but not from ΔAARE (N = 12/group) or OGH (N = 9/group) reporters. **C.** HepG2 cells were transfected with reporter vectors OGH, AARE-WT, ΔAARE, ΔA or ΔC, along with 0 ng (dark grey bars), 125 ng (light grey bars) or 250 ng (white bars) of ATF4 expression plasmid, with increasing amounts depicted by the wedges. The number of replicates per group is shown below each bar. Expression from AARE-WT reporter without recombinant ATF4 was considered 100%; expression from the other groups are shown in comparison to this. Note the break in the Y-axis at 800%; expression from WT vector with ATF4 is graphed on the upper portion, all other groups on the lower portion. All effects of ATF4 coexpression were significant (p<0.001).

To test this hypothesis, the responsiveness of the endogenous *F7* gene to ATF4 was modeled in a reporter gene system. Reporter constructs containing a fragment of the human *F7* gene extending spanning the AARE were prepared in native and block-mutated configurations. The unmutated AARE has base sequence 5′ ATTTCATCA 3′, and like other identified AAREs appears to be a composite element with one half-site (residues –8 to –4, 5′ ATTTC 3′) resembling a C/EBP monomer consensus juxtaposed to a second half-site (residues –3 to +1, 5′ ATCA 3′) resembling an ATF monomer consensus. The block-mutated (ΔAARE) construct, with sequence 5′ ATGAGCGCA 3′, has base changes introduced at positions −2 through −6 to disrupt both half-site motifs. These plasmids, along with a promoterless control plasmid, were then used for various transient tranfection assays in HepG2 cells to assess responses to pre-existing endogenous ATF4 under the standard 5 mM glucose condition (Figure 7A), to induction of endogenous ATF4 by glucose deprivation (Figure 7B), and to overexpression of recombinant human ATF4 (Figure 7C).

In Figure 7A, HepG2 cells were cotransfected with the AARE-WT, ΔAARE, or promoterless control pOGH plasmids, along with either negative control siRNA or ATF4 siRNA, and cultured at the standard 5 mM glucose concentration. Reporter expression from the promoterless vector, as well as from the block-mutated vector, were not significantly changed by the presence of the ATF4 siRNA compared to the presence of the negative control siRNA. However, the extent of reporter expression from the native construct was reduced in the presence of the ATF4 siRNA. This reduction was significant although its increment was small, from 100+/−7.8% to 81.8+/−8.5%, p<0.001. In Figure 5B, the same reporter constructs were transfected into HepG2 cells prior to 24 hr treatment with 25 mM, 5 mM or 0 mM glucose. While reporter expression from the native construct increased significantly due to glucose deprivation (from 100+/−9.2% to 228+/−56%, p<0.001), expression from the promoterless control and ΔAARE constructs were unaffected.

In the experiments of Figures 7A and 7B, the unmutated *F7* reporter gene recapitulated the responses of the endogenous *F7* gene to ATF4 silencing under both ambient and glucose deprivation conditions. In contrast, the block-mutated reporter gene was inert both to reduction of ambient ATF4 by ATF4 siRNA and to induction of ATF4 by glucose deprivation, identifying the AARE as the promoter region required for the responses of the *F7* gene to ATF4.

In Figure 7C, the responsiveness of the native, block-mutated, and half-site mutated reporter constructs to overexpression of recombinant human ATF4 was assessed. For the native construct, the dose-responsive increase in reporter expression to recombinant ATF4 was robust. However, installation of double point mutations within either the ATF-motif (ΔA, with changes at positions −2 and −3), or the C/EBP-motif (ΔC, with changes at positions −6 and −7), as well as the block mutation affecting both motifs, severely impaired the influence of recombinant ATF4 on reporter expression. While all three mutated constructs responded weakly to recombinant ATF4, they were severely impeded in the extent of reporter upregulation in comparison to the construct with an unmutated AARE. Taken together, the data of Figure 5 confirmed that the effect of ATF4 on *F7* expression occurred through the AARE in its promoter, and indicated that mutation within either half-motif compromised the ability of the AARE to function.

### ATF4 Binds the *F7* AARE

**Figure 8:**
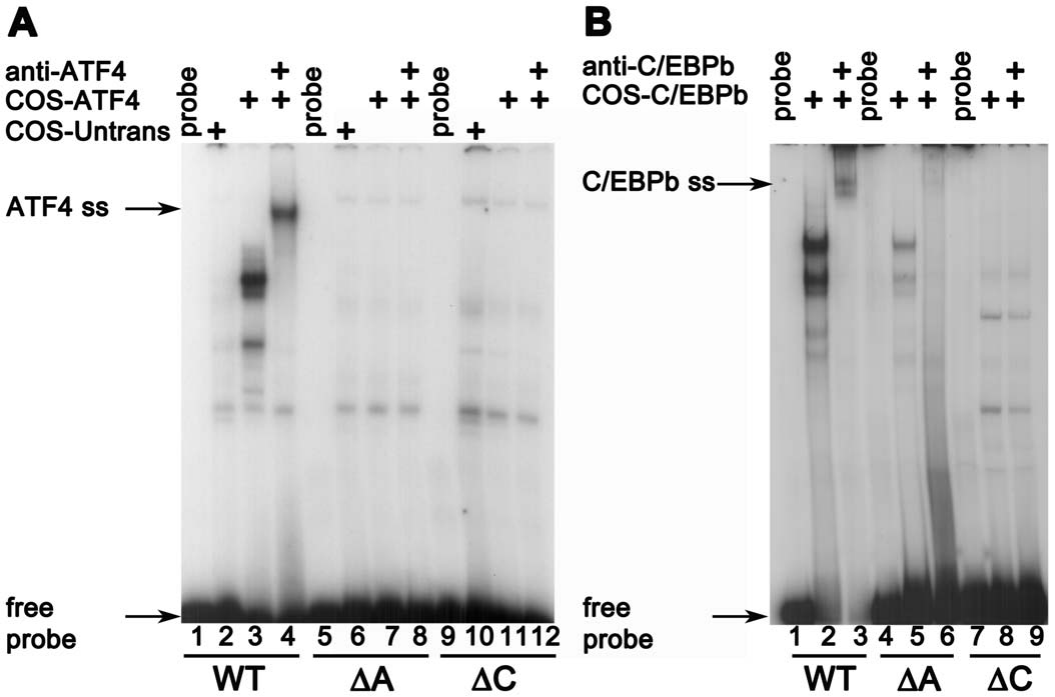
Recombinant ATF4 and C/EBPβ bind the F7 AARE. A. Oligonucleotides spanning F7 AARE without mutation (WT) or with mutation in the ATF-motif (ΔA) or the C/EBPβ-motif (ΔC), were incubated with 8 µg extracts of COS-1 cells that were untransfected, or transfected with expression plasmid for human ATF4. Extract used in each lane, and the lanes in which anti-ATF4 antibody were included, are indicated. The arrow indicates supershifted complex (ATF4 ss) detected in presence of antibody. **B.** A similar experiment, but using extract of COS-1 cells transfected with expression plasmid for human C/EBPβ in the absence or presence of anti-C/EBPβ antibody. The arrow indicates position of the supershifted complex (C/EBPβ ss).

The ability of ATF4 as well as C/EBPβ to directly bind to AARE-spanning oligonucleotides was explored by EMSA with recombinant human proteins overexpressed individually in COS-1 cells. As shown in Figure 8A, COS-1 cell extracts containing recombinant ATF4 produced a complex with the AARE-WT oligonucleotide, which was blocked and/or supershifted by an anti-ATF4 antibody. Neither the ΔA nor the ΔC oligonucleotide, however, was able to interact with ATF4. COS-1 cell extracts expressing human C/EBPβ were tested similarly, and the results are shown in Figure 8B. Two complexes of differing mobilities were observed binding to the unmutated *F7* oligonucleotide. Because the intronless C/EBPβ mRNA has three alternative initiation codons which can be selected by the cellular transcriptional machinery to produce the isoforms LAP* (or LAP-1), LAP (LAP-2), and LIP, a mixture of isoforms was expected. LAP* is the full-length protein; the slightly smaller LAP lacks only the N-terminal 23 amino acids. The truncated isoform, LIP, has DNA binding and dimerization domains identical to the longer isoforms, but lacks the entire N-terminal transactivation domain that they both possess [66]. Both C/EBPβ binding complexes observed in EMSA were blocked/supershifted by a pan-C/EBPβ antibody recognizing all isoforms. While weak residual binding of C/EBPβ-containing complexes was retained by the ΔA oligonucleotide, none was detectable with the ΔC oligonucleotide.

**Figure 9:**
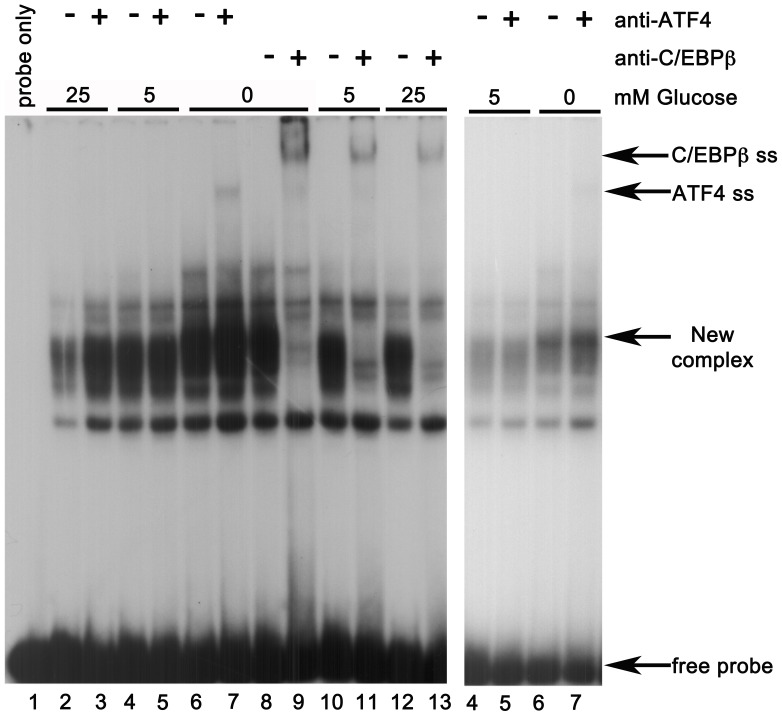
ATF4-containing complexes from glucose-deprived HepG2 cells bind F7 AARE. In each lane, 10 µg of nuclear extracts from cells cultured with 25 mM, 5 mM, or 0 M glucose interacted with AARE-WT probe, in the absence and presence of anti-ATF4 or anti-C/EBPβ antibodies. The darker exposure is shown at left; an ATF4 supershifted complex in lane 7 is indicated by arrow (ATF4 ss). Two C/EBPβ supershifted complexes are seen in lanes 9 and 11, one of which has identical mobility to ATF4 ss and the other which is indicated by arrow (C/EBPβ ss). In the lighter exposure of lanes 4 through 7, shown at right, a binding complex with slightly increased mobility in the 0 mM glucose condition (lanes 6 and 7) is more easily distinguished from the complexes of lanes 2–5 and indicted by arrow (new complex).

These data indicated that ATF4 as well as C/EBPβ bound the *F7* AARE, with a requirement that both half-motifs be intact for optimal binding to each transcription factor. The ability of endogenous ATF4 in nuclear extracts of HepG2 cells to interact with the AARE-WT oligonucleotide was examined next. As shown in the EMSA of Figure 9, glucose deprivation increased the overall detection of binding complexes and of a complex with slightly altered electrophoretic mobility. This complex is more easily discerned in the lighter exposure of lanes 1 though 7 shown on the right side of the figure, which is ∼ 20% of the intensity in the longer exposure shown on the left. Inclusion of antibody to ATF4 produced a supershifted complex with components of the nuclear extract prepared from glucose-deprived cells (lane 7), while inclusion of antibody to C/EBPβ blocked/supershifted the majority of complexes binding to the AARE-WT oligonucleotide under all glucose concentrations. The mobility of one complex supershifted by the anti-C/EBPβ antibody matched that of the complex supershifted by the anti-ATF4 antibody, and was most prominent in lane 9, containing nuclear extract prepared from glucose-deprived cells. This same supershifted complex is also detectable in lane 11, using nuclear extracts prepared from cells incubated with 5 mM glucose.

The data of Figure 9 suggested that at least some binding complexes from HepG2 nuclear extracts were heterodimeric species comprised of one ATF4 monomer and one C/EBPβ monomer. However, we also considered an alternative possibility, that individual binding complexes were composed of homodimers of either ATF4 or C/EBPβ isoforms, with one monomer binding optimally to its preferred half-site in the AARE and the other monomer interacting weakly but adequately with the non-preferred half-site. These alternatives are testable using mixing and supershift assays, provided the antibodies used do not cross-react significantly. To confirm that the antibodies were sufficiently specific for these experiments, reciprocal Western blotting of COS-1 cell extracts individually overexpressing recombinant human ATF4 or C/EBPβ was first performed. The results, presented in Figure S2, demonstrated that each antibody sensitively recognized the recombinant protein against which it had been raised. There was no indication that the anti-ATF4 antibody recognized C/EBPβ or that the anti-C/EBPβ antibody recognized ATF4. However, longer exposures of the blots showed that COS-1 cells also contained a protein recognized by the anti-C/EBPβ antibody, suggesting that these cells express a similar protein with nearly the same mass as human C/EBPβ. This cross-reacting protein can be seen faintly in lanes 1 and 2 of the anti-C/EBPβ blot of Figure S2.

**Figure 10:**
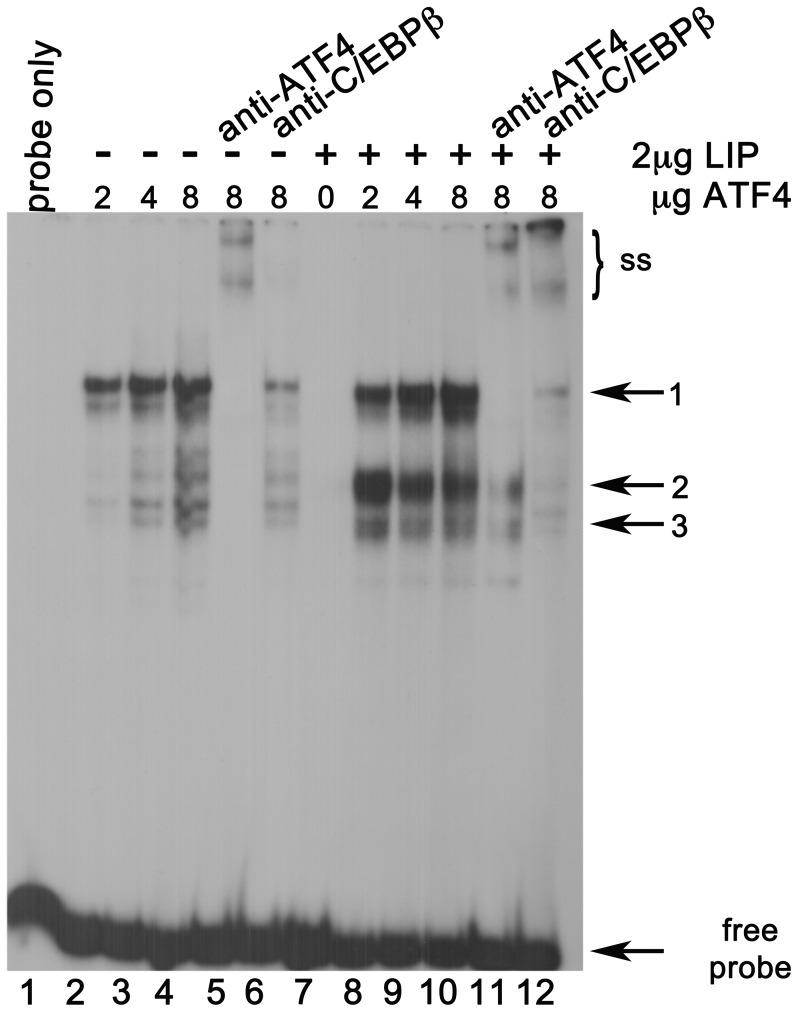
AARE binding species include ATF4-C/EBPβ heterodimers. WT oligonucleotide probe spanning the F7 AARE was titrated with 2 to 8 µg of extract from COS-1 cells overexpressing recombinant human ATF4, in the absence (-) or presence (+) of 2 µg of extract from COS-1 cells overexpressing recombinant human LIP. Blocking/supershift of binding complexes with anti-ATF4 and C/EBPβ antibody are shown without LIP in lanes 5 and 6 and with LIP in lanes 11 and 12. The bracket (ss) indicates the region of the gel with supershifted complexes. 2 µg of LIP extract did not produce a detectable complex when tested alone (lane 7).

Because mobility of oligonucleotide-protein complexes in EMSA is primarily determined by the mass of the protein constituents, we therefore performed the mixing experiment with recombinant human ATF4 and LIP, the smallest of the C/EBPβ isoforms. (Figure 10) ATF4-LIP heterodimeric complexes would be distinguishable from other potential dimeric complexes by migration pattern as well as by antibody recognition, since LIP monomers (∼20 kDa) are dissimilar in mass compared to ATF4 (∼50 kDa), LAP*/LAP (∼45 kDa each) and the C/EBPβ cross-reacting protein from COS-1 cells. Figure 10 shows results obtained when recombinant ATF4 or LIP were tested with the AARE-WT oligonucleotide, individually and in combination. A major complex of slow mobility (indicated by arrow 1) was observed on the AARE-WT oligonucleotide when titrated with 2–8 µg ATF4 extract. This complex was supershifted/blocked completely by the anti-ATF4 antibody, and partially by the anti-C/EBPβ antibody. The 2 µg of LIP extract used in this experiment was insufficient to produce a detectable complex at this exposure level when tested individually (lane 7). However, mixing of this amount of LIP extract with the titrated amounts of ATF4 extract produced two strong, readily detectable complexes of rapid mobility (arrows 2 and 3) and also intensified the original band that had been seen with ATF4 extract alone (arrow 1). Antibody directed towards either ATF4 or C/EBPβ supershifted/blocked all three complexes. Because combination of the two extracts synergistically increased total binding to the AARE-WT oligonucleotide and also produced prominent complexes of faster mobility (hence smaller mass) that were recognized by both antibodies, these data confirm that ATF4-C/EBPβ heterodimers are a *F7* AARE binding species. We conclude that increased expression of both ATF4 and C/EBPβ, and their subsequent interactions with the AARE of the *F7* gene, are responsible for upregulated *F7* transcription and FVII secretion during glucose deprivation.

## Discussion

Blood coagulation is an important process that maintains the integrity of the vascular system following injury. Coagulation is controlled on multiple levels to restrict its location and extent to sites of injury to the blood vessel wall. A major point of regulation of coagulation is control over the first reactions of the pathway, the interaction of FVII with its obligate receptor, TF. In order to initiate the complex process of blood coagulation, both components are not only required to come into contact but also to be activated. Unlike TF, which is synthesized by many cell types, is relatively abundant and is found primarily outside of the bloodstream, FVII is synthesized in trace amounts by hepatocytes and is found primarily within the bloodstream. Some FVII, however, apparently extravasates from the circulatory system and binds to TF in perivascular tissues. [67] Reduction of plasma FVII, such as may result from hereditary defects in the *F7* gene, cause a bleeding disorder of variable severity. When FVII levels are drastically reduced, the consequences of bleeding episodes can be life threatening. As a protein with a low plasma concentration and very short half-life, FVII must be consistently transcribed, synthesized and secreted in order to maintain plasma levels within an acceptable range for hemostasis.

Much work has shown that cellular stress conditions affect the ability of cells to synthesize secreted proteins. One well-characterized response to stressors including nutrient deprivation is repression of global protein synthesis, which reduces the energetic demands on the cells at a time when their resources are limited. In the present study, which investigated the effects of glucose deprivation on expression of coagulation protein expression, we found that several coagulation protein genes behaved in just that manner. As might be expected, their steady-state mRNA levels declined after 24 hr of glucose deprivation. However, one coagulation protein gene, encoding FVII, behaved in the converse manner. The work presented here demonstrated that *F7* expression (at both the mRNA and secreted antigen levels) was significantly increased in cells deprived of glucose. In contrast to the downregulation of expression of several other coagulation proteins, the upregulation of *F7* expression under glucose deprivation resembled that of the stress responsive transcription factors *ATF3*, *ATF6*, *C/EBPβ, XBP1(S)* and *CHOP*, as well as of the enzyme *ASNS*, the chaperone protein *GRP-78*, and the PP1 accessory factor *GADD34*. Previous work has examined the expression of recombinant FVIII cDNA in non-hepatic cell lines [68], [69], [70] and demonstrated that biosynthesis and secretion of the large FVIII protein, which carries numerous post-translational modifications, places taxing energetic demands even on cells that are highly specialized for protein secretion. We found that expression of endogenous *F8* was downregulated in HepG2 cells during glucose deprivation, as were *F10*, *F2*, and *PROS1*, all contrary to *F7*. To our knowledge, this was the first investigation of endogenous coagulation protein expression during cellular stress, and indicated that *F7* is regulated in an opposite manner to the others under our experimental conditions.

We found that glucose deprivation elevated both the steady-state *F7* mRNA within HepG2 cells, as well as the amount of FVII antigen secreted by them. Limitation of glucose was also associated with upregulation at both the mRNA and protein levels of transcription factor ATF4, and RNA interference experiments demonstrated that upregulation of *F7* mediated by glucose deprivation was causally related to the upregulation of *ATF4* as well as of *C/EBPβ*, an ATF4 target gene during cellular stress responses. [45], [71], [72] Although a supporting role for C/EBPβ, which itself interacts with the *F7* promoter [63] could not be ruled out, it was clear that the ATF4 upregulation was ultimately responsible for the increased endogenous *F7* expression observed during glucose deprivation. In an analogous manner, glucose deprivation increased the expression of a reporter gene directed by a fragment of native *F7* promoter, and mutational analyses of that fragment identified the amino-acid response element (AARE) as the region responsible for increasing *F7* reporter expression. Lastly, EMSA performed with nuclear extracts from cells deprived of glucose for 24 hr indicated that while the majority of AARE binding complexes contained C/EBPβ isoforms, a fraction also included ATF4. Mixing and supershift experiments using recombinant ATF4 and the truncated C/EBPβ isoform LIP, proteins of distinctly different masses, indicated that ATF4–C/EBPβ heterodimers can be formed on the AARE-WT oligonucleotide. Unexpectedly, in the aggregate, these data indicated that the F7 gene is a direct as well as indirect target of ATF4 in glucose-deprived cells. The *F7* gene directly interacts with and is transcriptionally responsive to ATF4, a master regulator of cellular stress responses.

The contribution of C/EBPβ to F7 upregulation during glucose deprivation cannot be overlooked. It should also be noted that, although C/EBPβ is strongly expressed in HepG2 cells even under non-stressed conditions, we found that siRNA directed against C/EBPβ reduced F7 expression under glucose deprivation. This was so, even though ATF4 induction was unaffected by the C/EBPβ siRNA, and though the overall extent of C/EBPβ expression still remained relatively high. Conversely, the siRNA directed against ATF4 had a greater impact on F7 expression during glucose deprivation than did the C/EBPβ siRNA, perhaps because it simultaneously diminished the upregulated expression of both ATF4 and C/EBPβ. These data imply not only that ATF4 and C/EBPβ cooperate in enhancing F7 expression, but that they do so most effectively under conditions when both are simultaneous upregulated. As has been suggested, [73] it is possible that newly-translated ATF4 and C/EBPβ will tend to dimerize with other newly-synthesized potential partners, making the formation of ATF4-C/EBPβ heterodimeric species more likely if both are being concurrently synthesized, but formation of homodimeric species more likely if synthesis of only one predominates at any given time.

Our data do not address whether all the endogenous C/EBPβ-containing binding complexes seen on EMSA are in heterodimeric complexes, or whether additional C/EBPβ binding partners besides ATF4 might be present at the *F7* AARE. Also, because the anti-C/EBPβ antibodies available for supershift analysis cannot discriminate between the transcriptional activators LAP*/LAP and the transcriptional inhibitor LIP, the data do not address which C/EBPβ isoforms are included. The broad band of C/EBPβ–containing complexes seen in all lanes of Figure 7 suggests that multiple complexes of slightly different masses exist within the cells and are capable of binding to the oligonucleotide. Whether those differences in binding complex size are due to the presence of different C/EBPβ isoforms, carriage of post-translational modifications, inclusion of additional binding partners, or to a mixture of causes, is unknown. A previous study of C/EBPβ expression during ER stress in rat C6 glioma cells has demonstrated that both the LAP and LIP isoforms are induced, but that their relative proportions depend not only on the period of stress but also on the nature of the stressor. [72] When stressed by a poison such as thapsigargin or tunicamycin, the intracellular levels of both LAP and LIP declined during the first three hours, then rebounded and were significantly elevated between nine and 24 hours. The ratio between LAP and LIP also changed, with LAP more prominent at the earlier times and LIP much more prominent at the later times. Interestingly, a different pattern was seen when cells were stressed in a more physiological manner. LAP and LIP were induced in tandem over twelve hours of amino acid deprivation and remained elevated to 24 hours; and the ratio between the isoforms was stable over this time course. The possibility that different stress conditions could produce subtle variations in the mechanism and outcome of the stress response itself is intriguing, and may have implications for the relative expression of numerous downstream target genes.

In our experiments, the period of stress and the nature of the stressor also influenced the outcome of coagulation factor expression in HepG2 cells. When 24 hr glucose deprivation was compared to 6 hr TG treatment, the *ATF4* and *C/EBPβ* genes were upregulated comparably. However, under glucose deprivation the expression of *F7* was elevated and expression of *F8* was reduced, while under TG treatment neither *F7* nor *F8* expression changed at all. Evidently, some critical difference in the cellular response to glucose deprivation versus TG treatment supports the different coagulation factor expression results obtained. Although the reason(s) for this discrepancy in coagulation factor expression is unclear, one caveat must be borne in mind: the C/EBPβ gene expression assay used in our experiments cannot distinguish among the isoforms. Therefore it is not known whether *C/EBPβ* upregulation under glucose deprivation versus TG treatment produced similar proportions of the isoforms LAP*/LAP and LIP.

ATF4 is induced to some degree under nearly all stress conditions. Frequently, increased ATF4 transcription and preferential ATF4 translation occur together, coordinating to have a large impact on the level of functional ATF4 transcription factor generated. [60], [73], [74], [75], [76] Rarely, transcription of ATF4 is found not to change or even to be reduced by a stressor. For example, ultraviolet irradiation decreases ATF4 transcription, and therefore ATF4 protein levels do not increase in UV-irradiated cells despite high eIF2α phosphorylation [34], [74]. An expanding list of ATF4 target genes have now been identified, and many have been shown to possess functional AAREs within their regulatory regions, which mediate their transcriptional upregulation during stress responses. Many of these ATF4 target genes are themselves transcription factors needed to carry out the altered gene transcription patterns of adaptive stress responses. Additional ATF4 target genes encode proteins that alleviate a localized stress in a straightforward manner. For example, genes encoding asparagine synthetase as well as the system 2 neutral amino acid and cystine-glutamate transporters, are induced in response to glucose, amino acid or protein limitation. [37], [41], [43] These genes are ubiquitously expressed and encode either intracellular or membrane proteins, rather than secreted proteins. By increasing the cellular capacity to synthesize amino acids or to acquire them from the environment, the biological roles of the induced enzymes/transporters are to directly counteract the nutritional stress being experienced by the responding cells.

In contrast, there are other ATF4 target genes whose expression is tissue-restricted, whose products are secreted from their cells of origin, and whose roles in response to stress conditions are less immediate or comprehensible. Such genes include osteocalcin, insulin growth factor binding protein 1 and vascular-endothelial growth factor. Osteocalcin is a calcium-binding component of bone matrix produced by osteoblasts, which also acts as a hormone for adipocytes and the pancreas. [77]. Insulin growth factor binding protein 1 is a member of a family of secreted proteins that regulate the activity of the insulin-like growth factors. Its biosynthesis is tissue-restricted; liver, kidney, and female reproductive organs are the major sites. [78], [79] Vascular-endothelial growth factor is a secreted protein expressed in response to low oxygen tension, which acts as a mitogen via interaction with endothelial cell surface receptors to promote and direct the growth of new blood vessels. [80], [81], [82] Expression of these particular stress-responsive target genes is required during various aspects of development, growth, and tissue repair, and an AARE capable of binding to ATF4 has been identified within a regulatory region of each. Although the ATF6 and IRE1α branches of the stress response were shown to contribute to upregulation of vascular-endothelial growth factor, [82] it is evident that ATF4-dependent mechanisms are primarily responsible for upregulation of these genes under stress conditions.

Our work indicates that *F7* is an additional example of a stress-inducible gene with tissue-restricted expression, which encodes a secreted protein having the potential to either maintain normal systemic function despite the stressor and/or to modulate systemic responses to the stressor. HepG2 cells stressed by lack of available glucose devoted a portion of their limited resources to the production of this coagulation protein, and then secreted it, sustaining a net loss of material that might otherwise have been directed to biochemical salvage pathways or to catabolism. It is not completely clear why, among the coagulation factor genes tested, expression of only the *F7* gene was transcriptionally upregulated. But considering its very short half-life in plasma in comparison to other coagulation proteins and its essential role as a co-initiator of blood coagulation along with TF, *F7* upregulation could plausibly be a compensatory mechanism to maintain adequate hemostasis during periods of stress. Reductions in the plasma concentrations of some procoagulant proteins, such as FX or prothrombin, with half lives of 40–70 hr, might be expected to have a lesser potential impact on hemostasis than reduction in the plasma concentration of FVII, with its half life of 3–6 hr.

Beyond its critical role in blood coagulation, the TF–FVIIa complex plays both direct and indirect roles in signal transduction processes of non-hepatic cells, which could also be affected by the plasma level of FVII. The capacity of monocyte TF, a transmembrane protein with similarity to class 2 cytokine receptors, to participate directly in signal transduction when stimulated by FVII binding has been investigated following LPS stimulation, and upregulated expression of pro-inflammatory cytokines such as IL-6, IL-8 and TNFα was observed. [83]In addition, when bound to TF on a cell surface, FVIIa has been shown to trigger signal transduction through cleavage and activation of the G-protein coupled receptor, protease-activated receptor 2. (reviewed in [84]) Some additional consequences of TF–FVII interactions, not all of which relate to hemostasis, may be inferred from the results of investigations performed with the “low-FVII” transgenic mouse, which expresses ∼1% of the average normal level of plasma FVII. On the one hand, the low-FVII mouse suffers slower wound healing, and is more prone to cardiomyopathy as a result of post-hemorrhage fibrosis within the heart. [85], [86] On the other hand, the low FVII mouse experiences reduced experimental asthmatic responses, and is more likely to survive LPS-induced endotoxemia. [87], [88] Comparison of the responses of the low-FVII mouse and its normal-FVII counterpart to various challenges thus suggests that the outcomes of the TF–FVII interaction may be alternately beneficial or detrimental, especially in response to inflammatory stimuli.

The data we present also showed that ATF4 steady-state mRNA and protein were present and functional in HepG2 cells even under nominally un-stressed conditions, indicating that a degree of eIF2α phosphorylation continuously but transiently takes place and then is resolved. Indeed, phospho-eIF2α was detected by Western blot in cells even without glucose deprivation. This is unlikely to be an idiosyncrasy of HepG2 cells, since it has been shown that feeding acutely increases activation of stress markers in the liver. [89], [90] Indeed, ATF4 protein is detectable in the livers of normal mice following a 12 hr fast, and is further induced by a 4 hour continuous nutrient infusion supplying approximately 50% of the typical daily caloric intake. [91].

That both fasting and feeding have both been linked to upregulated ATF4 in hepatic cells from healthy animals, albeit to different extents and perhaps with other permutations as well, is intriguing. Such ATF4 oscillations may be transient in healthy individuals but more sustained, or of different character, in those with metabolic disorders affecting the liver that develop insidiously such as diabetes. [90], [92] Activation of ER stress response pathways has been linked with establishment of hepatic insulin resistance, which is a hallmark of diabetes, while alleviation of that stress has been correlated with restoration of insulin sensitivity. [93], [94] In this regard, it is interesting to note that plasma levels of coagulation proteins, including FVII, are often elevated in diabetics [95] and that our previous work demonstrated downregulation of *F7* expression by insulin treatment. [63] In the insulin-resistant state, the loss of such negative regulation of gene expression would passively permit increased *F7* transcription to take place. However, the work presented here implies that increased ATF4-dependent transcription during the cellular stress elicited by insulin resistance might comprise an additional positive regulatory mechanism contributing to elevated plasma FVII in diabetics. The physiological implications of increased *F7*/FVII expression resulting from glucose deprivation, or other stressors provoking ATF4 induction, are deserving of further attention considering the plieotropic effects of TF–FVII interactions.

## Materials and Methods

### Cell Culture and Treatments

The human hepatoma cell line, HepG2 (HB-8065), was obtained from the American Type Culture Collection (Manassas, VA) and routinely cultured in full growth medium as described described [63]. For experimental treatments, 2×10^5^ cells were plated per well in 6-well dishes, cultured to approximately 70% confluence in full growth medium, then transferred for an additional 24 hr to collection media, consisting of glucose-free D-MEM (Sigma-Aldrich, St Louis, MO) containing 10% or 1% (v/v) fetal bovine serum, and supplemented with D-glucose (Life Technologies, Carlsbad, CA) to the desired final concentration. Aliquots of the conditioned collection media were assayed for secreted factor VII antigen by enzyme-linked immunoabsorbent assay (ELISA) (Enzyme Research Labs, South Bend, IN). HepG2 whole cell lysates were prepared for Western blotting using RIPA lysis and extraction buffer supplemented with HALT Protease and Phosphatase Inhibitor cocktail (Thermo Fisher Scientific, Rockford, IL). Thapsigargin (Calbiochem EMD Millipore, Billerica, MA) at 500 nM final and Sal003 (Santa Cruz Biotechnology, Santa Cruz, CA) at 10 µM final were added to cultures for 6 hr where indicated. Nuclear extracts were prepared by hypotonic lysis described [63] for use in EMSA. COS-1 cells (ATCC CRL-1650) were routinely cultured in Dulbecco's modified Eagle's medium (D-MEM; Life Technologies) plus 10% serum supplemented with 100 U/ml penicillin G, 100 mg/ml streptomycin at 37°C in an atmosphere of 5% CO_2._ Cell numbers were determined by trypsinization and hemocytometer counting in duplicate for individual replicate cultures. Metabolic activity was indicated with the Cell-Titer assay (Promega, Madison, WI) performed according to the manufacturer’s protocol. Additional details for the Cell-Titer experiment are provided in Methods S1.

### Reporter Constructs and Expression Plasmids

A human growth hormone (hGH) reporter plasmid containing a segment of native *F7* 5′ flanking sequence extending from position −728 to +134 relative to the translation start site of the gene (with the initiation codon at positions +1 to +3), was constructed in promoterless plasmid pOGH by PCR as described [63]. Plasmids having block-mutated *F7* AARE sequence (ΔAARE, 5′ AT**GAGCG**CA 3′), and half-site mutated sequences affecting only the ATF motif (ΔA, 5′ ATTTC**CG**CA 3′) or only the C/EBP motif (ΔC, 5′ A**AG**TCATCA 3′) were made by overlapping PCR using the native construct (WT, 5′ ATTTCATCA 3′) as the template. The AARE sequences from positions −8 to +1 are given, with mutated bases shown in bold type. All plasmids were confirmed by DNA sequencing on both strands. The expression vector for human C/EBP-LIP was the gift of Dr. M. Kilberg (Gainsville, FL), while those for full-length human C/EBPβ and for ATF4 were obtained from Origene Technologies (Rockville, MD).

### Transfections

Transfection of HepG2 cells with hGH reporter plasmids and β–galactosidase plasmid as a transfection efficiency control were performed using Attractene (Qiagen Inc., Germantown MD) or Lipofectamine (Life Technologies) according to the manufacturers’ protocols for this cell line. With Attractene, 3×10^5^ cells per well were transfected in full growth medium at plating with 800 ng of reporter vector and 200 ng of pRSV-β–galactosidase expression vector. Approximately 40 hr later, the growth medium was replaced with collection medium for an additional 24 hr prior to harvest. Conditioned media and cell lysates were respectively assayed for hGH by ELISA (Diagnostic Systems Labs, Webster, TX; or Roche Applied Science, Indianapolis, IN) and for β–galactosidase by colorimetric enzyme assay (Promega). Attractene reagent was used for all DNA transfections except those shown in Figure 7C, which were done with Lipofectamine reagent. In those experiments, 1×10^6^ cells per well were plated in full growth medium and transfected 18 hr later with a total of 4 µg of DNA. 2 µg of reporter vector, 0.5 µg of pRSV-β–galactosidase vector, and 125 ng or 250 ng of human ATF4 expression plasmid were introduced, with the total amount of DNA equalized by addition of the inert plasmid, pUC19.

In all reporter gene assays, six replicates per reporter vector were routinely done per experiment. Transfection data were expressed as average percentage of normalized WT reporter expression, plus or minus the standard deviation (SD). The statistical significance of differences in reporter expression between groups was assessed by the Student's t-test.

5 nM small interfering RNA (siRNA) was introduced into 2.5×10^5^ HepG2 cells at plating using Hi-Perfect transfection reagent (Qiagen) according to the manufacturer’s protocol. Cells were cultured in full growth medium for approximately 40 hr before transfer to collection medium for the final 24 hr incubation prior to harvest. Six replicates were transfected per experiment, with cells from pairs of wells combined for RNA preparation. The siRNAs directed against human ATF4 (SI03019345 and SI03218404) and human C/EBPβ (SI02777292) were obtained from Qiagen and used for targeted knockdowns. Negative and positive transfection controls, the All-Stars negative siRNA and All-Stars Death siRNA (also from Qiagen) were included in siRNA experiments. The statistical significance of differences between groups transfected with negative control siRNA and transcription factor siRNA were assessed by the Student's t-test.

For preparation of extracts overexpressing recombinant human ATF4, C/EBPβ or LIP, approximately 10×10^6^ COS-1 cells were transfected with 10 µg of expression plasmid using Lipofectamine reagent. The cells were cultured in full growth medium for 48 hr, when whole-cell lysates were prepared by a freeze-thaw method. [96].

### Electrophoretic Mobility Shift Assay (EMSA)

Complementary oligonucleotides extending from position −20 to +25 of the *F7* gene were annealed and end-labeled with [γ−^32^P]-ATP (Perkin-Elmer Life Sciences, Billerica, MA) using T4 polynucleotide kinase (New England Biolabs, Beverly MA). Binding reactions were done in buffer containing 5 mM HEPES pH 7.5, 30 mM KCl, 3 mM MgCl_2_, 0.5 mM EDTA, 0.5 mM dithiothreitol, 12% glycerol, and 1 µg of poly (dI-dC). Incubations of HepG2 nuclear extracts or COS-1 whole cell extracts with radiolabeled probe were carried out for 15 minutes at room temperature. For supershift, 60 min preincubation of extract in binding buffer with antibody was done at 4°C preceded the addition of probe. Reactions were electrophoresed on 5% (w/v) polyacrylamide gels in tris-borate-EDTA buffer (90 mM Tris base, 90 mM sodium borate, and 0.5 mM EDTA) at 300 volts for 3 hr with cooling to 4°C, then dried and autoradiographed.

For mixing experiments, COS-1-LIP whole-cell extract was diluted in binding buffer and mixed with titrated amounts of COS-ATF4 whole-cell extract for a 60 min preincubation period, followed by probe addition and an additional 15 min incubation period prior to electrophoresis. For supershift assays, polyclonal antibodies to ATF4 or C/EBPβ (sc-200X and sc-150X, Santa Cruz Biotechnology) were added to reaction mixtures at the start of the preincubation period.

### Western Blotting

Whole-cell extracts of HepG2 cells were prepared following experimental treatments as described, and of COS-1 cells at 48 hr post transfection with recombinant expression vectors, respectively. The protein concentrations were determined by the BCA protein assay (Pierce Biotechnology, Rockford IL). Aliquots were denatured in standard Laemmli sample buffer (Sigma, St. Louis MO), separated on 4–20% discontinuous polyacrylamide gels (ThermoFisherScientific) at 150 volts for 45 min at room temperature, transferred to Immobilon membranes (Life Technologies) at 20 volts for 2 hr at 4°C, blocked overnight with 5% (w/v) dry milk and 5% (w/v) bovine serum albumin, probed 2 hr with appropriate primary antibody then 1 hr with horseradish peroxidase-conjugated secondary antibody (Santa Cruz; Calbiochem, San Diego, CA), and developed with Dura-Signal West chemiluminescent reagents (Pierce) at room temperature. Primary antibodies to ATF4 and C/EBPβ were the same as those used for EMSA; antibodies to CHOP, ATF3, XBP1 (S), pan-eIF2α and GAPDH were from Santa Cruz, while antibody to phospho-eIF2α was from Cell Signaling Technologies (Beverly, MA). Experimental assessment of cross-reaction potential of the anti-ATF4 and anti-C/EBPβ antibodies is described in Methods S1.

### Quantitative Reverse-transcriptase Realtime PCR

Total RNA was prepared by the RNeasy miniprep protocol (Qiagen), and converted to cDNA using the High-Capacity RNA to cDNA kit (Applied Biosystems, Foster City CA). Aliquots of cDNA were tested in qRT-PCR using Taq-Man gene-specific assay mixtures in an Applied Biosystems 7900 detector under standard cycling conditions of 2 min at 48°C, 10 min at 95°C, followed by 40 cycles of PCR with 15 sec at 95°C/1 min at 60°C. This procedure was used to monitor relative expression of various genes (*ATF4*, *C/EBPβ, ASNS, ATF3, GRP-78,CHOP, GADD34, ATF6, XBP1(U), XBP1(S), F7, F8, FX, F2, PROS1;* catalogue numbers of gene expression assays given in Table S1) normalized against expression of the 18S ribosomal RNA gene, by the ΔΔCt method. All values are percent expression +/− SD. Unless otherwise noted, the number of replicates per condition was three per experiment, with each sample assayed in duplicate for each amplicon. Statistical difference between groups was assessed by the student’s t-test. qRT-PCR was also performed with two types of control assay. Conversion reactions from which the reverse transcriptase enzyme had been omitted were done to detect DNA contamination of the RNA preparations, and no-template controls were performed for each gene-specific assay. The controls were always negative.

## Supporting Information

Figure S1 **Cellular characteristics.** At left, cells were plated on a 96-well dish, at cell numbers and culture medium volumes proportional to those used in all other experiments. Collection media with 5 mM or 0 mM glucose were applied at the usual time, and the CellTiter reagent (Promega) was included and assayed according to the manufacturer’s protocol. N = 8 per group, p<0.001. At right, a parallel experiment was set up on 6-well dishes with proportional initial cell numbers and media volumes. At the final time, cells were trypsinized and counted using a hemocytometer. The average of 8 cell count determinations per well are shown; the difference between the 5 mM and 0 mM glucose groups was not significant.
(TIF)

Figure S2 **Antibody specificity.** 20 µg aliquots of extracts from untransfected COS-1 cells, or cells individually overexpressing recombinant human ATF4 or human C/EBPβ (as shown above lanes) were separated by SDS-PAGE and subjected to Western blotting with anti-ATF4 or anti-C/EBPβ antibody as shown to the right of each panel. As loading controls, replicate samples were blotted with anti-GAPDH antibody.
(TIF)

Table S1 **Gene expression assays.** The target genes used for RT-PCR experiments are shown in the first column. The genbank accession numbers, and Applied Biosystems/Life Technologies gene expression assay numbers, are given for each target gene.
(DOC)

Methods S1 **Detailed methodology for the experiments shown in Figures S1 and S2 are provided.**
(DOC)
