# Supplementary figures and images for: Upregulation of the Coagulation Factor VII Gene during Glucose Deprivation Is Mediated by Activating Transcription Factor 4
Source: PLoS One. 2012 Jul 27;7(7):e40994. doi: 10.1371/journal.pone.0040994 (PMC3407153; doi:10.1371/journal.pone.0040994)

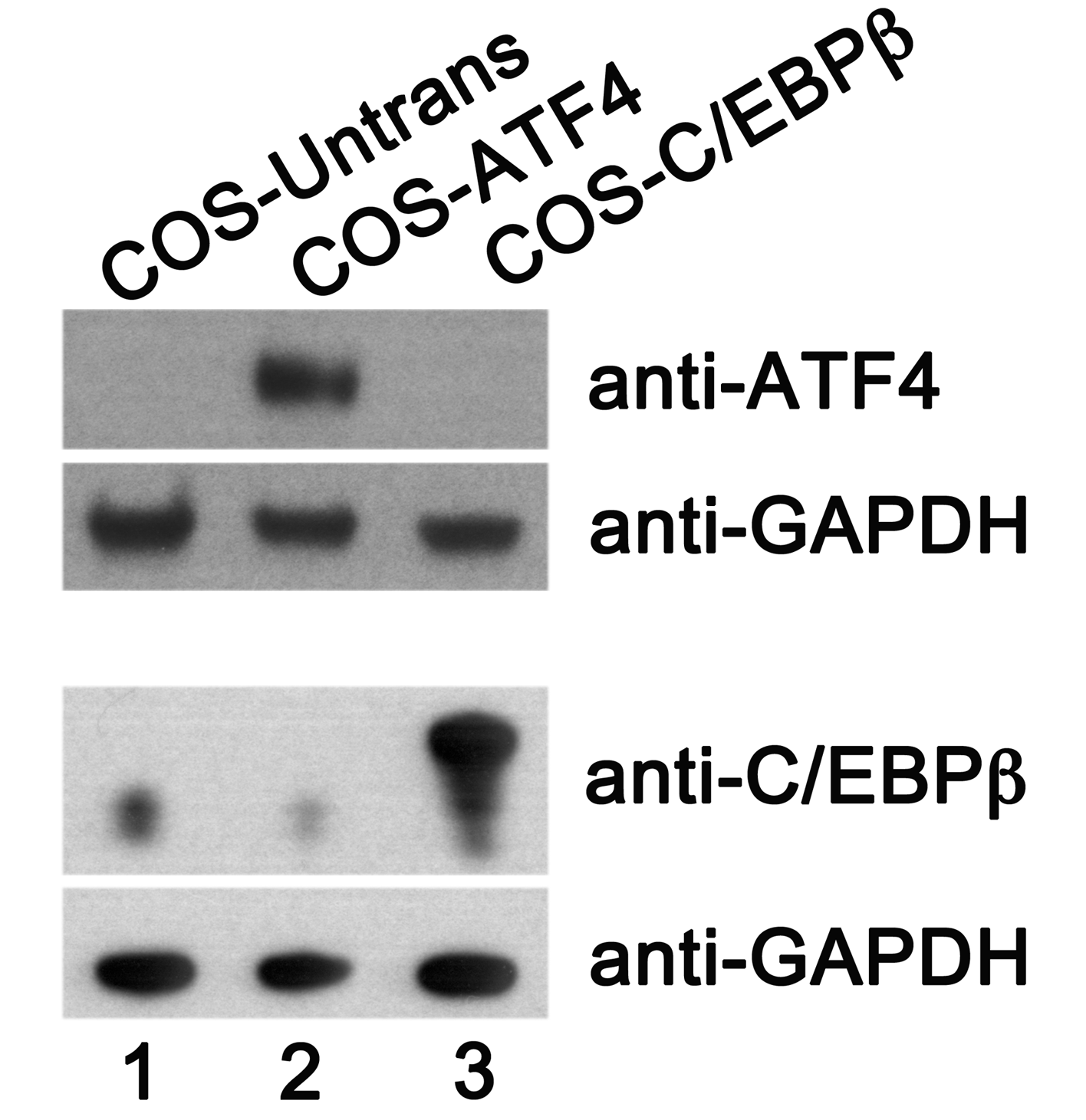

Supplement: Figure S1 — Cellular characteristics. At left, cells were plated on a 96-well dish, at cell numbers and culture medium volumes proportional to those used in all other experiments. Collection media with 5 mM or 0 mM glucose were applied at the usual time, and the CellTiter reagent (Promega) was included and assayed according to the manufacturer’s protocol. N = 8 per group, p<0.001. At right, a parallel experiment was set up on 6-well dishes with proportional initial cell numbers and media volumes. At the final time, cells were trypsinized and counted using a hemocytometer. The average of 8 cell count determinations per well are shown; the difference between the 5 mM and 0 mM glucose groups was not significant. (TIF) [file pone.0040994.s001.tif]

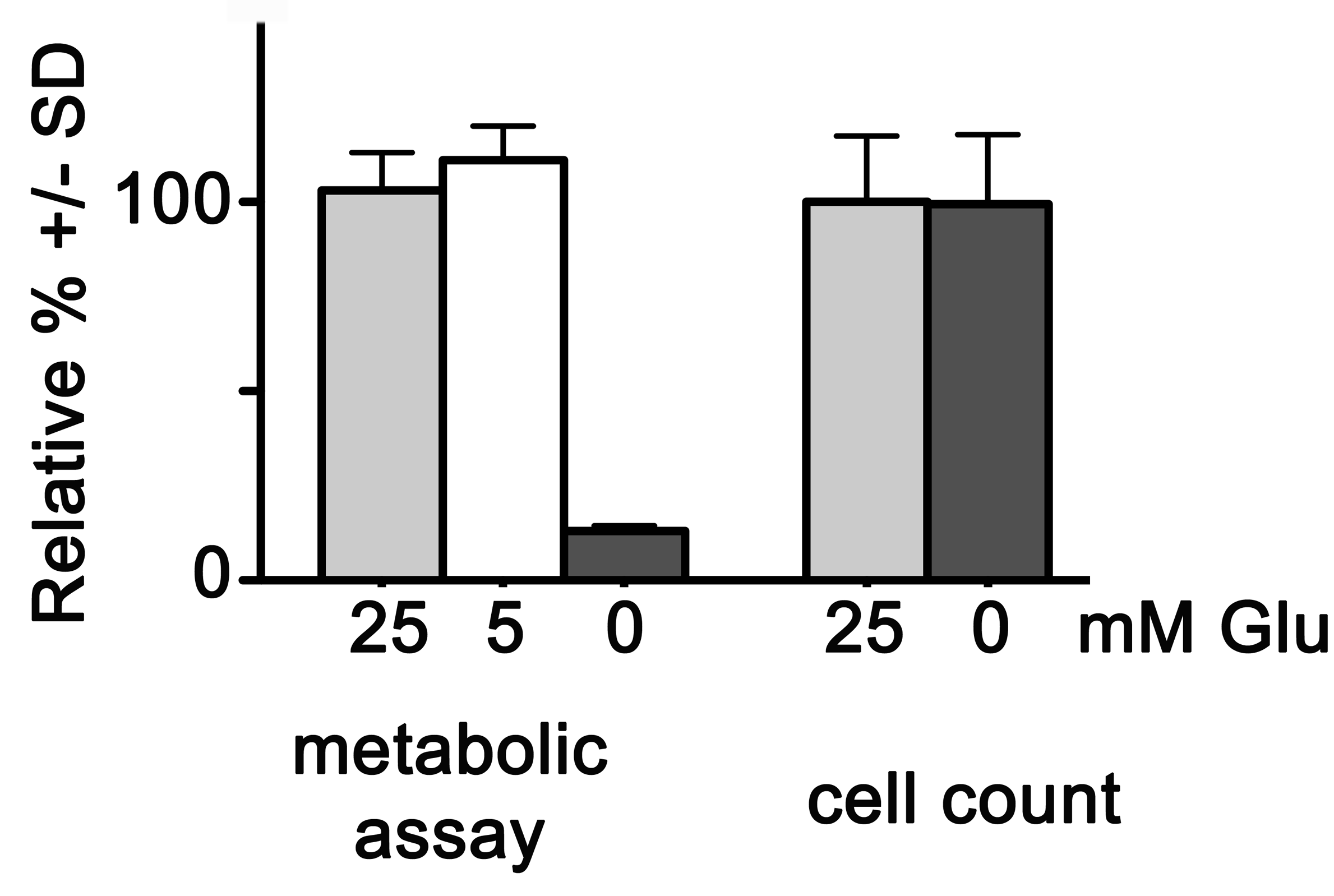

Supplement: Figure S2 — Antibody specificity. 20 µg aliquots of extracts from untransfected COS-1 cells, or cells individually overexpressing recombinant human ATF4 or human C/EBPβ (as shown above lanes) were separated by SDS-PAGE and subjected to Western blotting with anti-ATF4 or anti-C/EBPβ antibody as shown to the right of each panel. As loading controls, replicate samples were blotted with anti-GAPDH antibody. (TIF) [file pone.0040994.s002.tif]
