# Supplementary material for: Upregulation of the Coagulation Factor VII Gene during Glucose Deprivation Is Mediated by Activating Transcription Factor 4
Source: PLoS One. 2012 Jul 27;7(7):e40994. doi: 10.1371/journal.pone.0040994 (PMC3407153; doi:10.1371/journal.pone.0040994)
Supplement: Table S1 — Gene expression assays. The target genes used for RT-PCR experiments are shown in the first column. The genbank accession numbers, and Applied Biosystems/Life Technologies gene expression assay numbers, are given for each target gene. (DOC) [file pone.0040994.s003.doc]

**Supplemental Table 1. Gene Expression Assays**

| **Gene Name** | **Accession Number** | **Assay Number** |
| --- | --- | --- |
| F7 | NM_01916 | HS00173398_m1 |
| FX | NM_000504 | HS00173450_m1 |
| F2 | NM_000506 | HS 01011988_m1 |
| F8 | NM_000132 | HS00252034_m1 |
| PROS1 | NM_00313 | HS00165590_m1 |
| ATF-4 | NM_001675.2,NM_877962.1 | HS00909569_g1 |
| ATF-3 | NM_00164 | HS00231069_m1 |
| ATF6 | NM_007348 | HS00232586_m1 |
| C/EBP beta | NM_005194 | HS00270923_s1 |
| XBP1(U) | NM_005080.3,NM_001079539.1 | HS00231936_m1 |
| XBP1(S) | NM_001079539.1 | HS03929085_g1 |
| ASNS | NM_001673 | HS00155888_m1 |
| GRP78 | NM_005347 | HS99999174_m1 |
| GADD34 | NM_014330 | HS00169585_m1 |
| DDIT3 (CHOP) | NM_001195053 | HS00358796_g1 |
| 18S rRNA |  | 4319413E |
