# Supplementary material for: Upregulation of the Coagulation Factor VII Gene during Glucose Deprivation Is Mediated by Activating Transcription Factor 4
Source: PLoS One. 2012 Jul 27;7(7):e40994. doi: 10.1371/journal.pone.0040994 (PMC3407153; doi:10.1371/journal.pone.0040994)
Supplement: Methods S1 — Detailed methodology for the experiments shown in Figures S1 and S2 are provided. (DOC) [file pone.0040994.s004.doc]

**Supplemental Methods**

**Cell culture and treatments.** The human hepatoma cell line, HepG2 (HB-8065), was obtained from the American Type Culture Collection (Manassas, VA). 2 x 10^5^ cells were routinely plated per well in 6-well dishes, cultured in full growth medium (minimal essential medium (MEM) with 5 mM glucose and 10% fetal bovine serum), to approximately 70% confluence, then transferred for an additional 24 hr to collection media, consisting of glucose-free D-MEM (Sigma-Aldrich, St Louis, MO) containing 1% (v/v) fetal bovine serum, and supplemented with D-glucose (Life Technologies, Carlsbad, CA) to the desired final concentration. Cell numbers were determined by trypsinization and replicate hemocytometer counting for each individual sample. Metabolic activity was indicated with the CellTiter assay (Promega, Madison, WI) performed according to the manufacturer’s protocol. 1 x 10^4^ cells were plated per well of a 96-well black cell culture assay plate (Costar, Corning NY), with 0.1 ml full growth medium and grown to ~70% confluence, then changed to collection medium with glucose as shown for 24 hr. The MTS 3-(4,5-dimethylthiazol-2-yl)-5-(3-carboxymethoxyphenyl)-

2-(4-sulfophenyl)-2H-tetrazolium reagent was added and absorbance at 490 nM was read, after 1 hr incubation in the cell culture incubator, on a Molecular Devices FlexStation3 plate reader.

**Transfections and Western blotting.** For preparation of extracts overexpressing recombinant human ATF4, C/EBPβ or LIP, approximately 10 x 10^6^ COS-1 cells were transfected with 10 µg of individual expression plasmid using Lipofectamine reagent. The ATF4 and C/EBPβ expression plasmids were obtained from Origene Technolgies (Rockville, MD), the LIP expression plasmid was provided by Dr Michael Kilberg (University of Florida, FL, USA). The cells were cultured in full growth medium for 48 hr, when whole-cell lysates were prepared by a freeze-thaw method. [96] The protein concentrations were determined by the BCA protein assay (Pierce Biotechnology, Rockford IL). Aliquots were denatured in standard Laemmli sample buffer (Sigma, St. Louis MO), separated on 4-20% discontinuous polyacrylamide gels (ThermoFisherScientific) at 150 volts for 45 min at room temperature, transferred to Immobilon membranes (Life Technologies) at 20 volts for 2 hr at 4^o^ C, blocked overnight with 5% (w/v) dry milk and 5% (w/v) bovine serum albumin, probed 2 hr with primary antibody then 1 hr with horseradish peroxidase-conjugated secondary antibody (Santa Cruz Biotechnology, Santa Cruz CA; Calbiochem, San Diego, CA), and developed with Dura-Signal West chemiluminescent reagents (Pierce, Rockford, IL) at room temperature. Primary antibodies to ATF4 (sc-200) and C/EBPβ (sc-150) were from Santa Cruz Biotechnology.
